# Programmable polymorphism of a virus-like particle

**DOI:** 10.1038/s43246-022-00229-3

**Published:** 2022-02-07

**Authors:** Artur P. Biela, Antonina Naskalska, Farzad Fatehi, Reidun Twarock, Jonathan G. Heddle

**Affiliations:** 1 Malopolska Centre of Biotechnology, Jagiellonian University, Gronostajowa 7A, 30-392 Krakow, Poland; 2 Departments of Mathematics, University of York, York YO10 5DD, UK; 3 York Cross-Disciplinary Centre for Systems Analysis, University of York, York YO10 5GE, UK; 4 Department of Biology, University of York, York YO10 5DD, UK

## Abstract

Virus-like particles (VLPs) have significant potential as artificial vaccines and drug delivery systems. The ability to control their size has wide ranging utility but achieving such controlled polymorphism using a single protein subunit is challenging as it requires altering VLP geometry. Here we achieve size control of MS2 bacteriophage VLPs via insertion of amino acid sequences in an external loop to shift morphology to significantly larger forms. The resulting VLP size and geometry is controlled by altering the length and type of the insert. Cryo electron microscopy structures of the new VLPs, in combination with a kinetic model of their assembly, show that the abundance of wild type (*T* = 3), *T* = 4, D3 and D5 symmetrical VLPs can be biased in this way. We propose a mechanism whereby the insert leads to a change in the dynamic behavior of the capsid protein dimer, affecting the interconversion between the symmetric and asymmetric conformers and thus determining VLP size and morphology.

Protein cages, convex polyhedral protein containers self-assembled from multiple copies of identical protein subunits, are pillars of nanotechnology. They include naturally occurring virus capsids (virus-like particles, VLPs) as well as a plethora of particles with diverse symmetries. Given their structures, protein cages are obvious candidates for development as vaccines (through addition of relevant antigens on their exterior surfaces) and as drug delivery systems (through encapsulation of relevant therapeutic molecules in their hollow cores)^1–3^. Additionally, VLPs have an innate ability to encapsulate nucleic acids, which makes them attractive as DNA/RNA delivery vehicles. However, to date, they have not been approved as therapeutic agents other than as vaccines. Two such vaccines (protecting against HBV and HPV infections) are currently approved for use in humans while many others are under clinical trial (reviewed in ref. ^4^).

The ability to control VLP particle size is attractive as it allows the same constituent protein components to be repurposed in VLPs having different morphologies and properties. Increasing VLP size is particularly beneficial, resulting in both greater surface area for increased antigen presentation and larger volume with increased cargo capacity. However, this is challenging to achieve given that VLPs are made from multiple proteins, each forming a number of distinct bonds with their neighbors. As a consequence, changing the number of subunits and the angle between them, as would be necessary to increase overall diameter, is difficult without disrupting the entire structure. Nevertheless, polymorphism is well established amongst naturally occurring viruses/VLPs^5,6^. It has been reported, for example, that the VLP derived from bacteriophage P22 can expand or contract as a result of heating to 65 °C or following treatment with sodium dodecyl sulfate, resulting in the opening and closing of pores in the particle which can be used for cargo entry or egress^7^. The SV40 virus capsid, formed from major capsid protein VP1, can switch between *T* = 7d, *T* = 1 and tubular structures depending on salt concentration and pH^8,9^. Lumazine synthase, a protein cage of bacterial origin, has also shown a similarly wide range of different forms, including cages of different sizes and even a protein tube depending on buffer conditions^10^. In all of these cases, the spectrum of variant morphologies, and how assembly can be biased to achieve desired structural outcomes, are unknown.

MS2 is an icosahedral RNA bacteriophage measuring 22–29 nm in diameter^11^. Its *T* = 3 capsid is built from 180 copies of a single coat protein (CP). The RNA genome (~3.6 kb) encodes a further three proteins: the maturation protein (A-protein), the replicase, and the lysis protein. When CP is expressed from a plasmid in a bacterial or yeast host, in the absence of other viral elements, it self-assembles into VLPs^12^, dimerizing in the process. An unstructured loop known as the FG-loop connects the F and G β-strands of each subunit. The 90 dimers forming the VLP can be classified into two structurally different groups: one group contains 60 asymmetric dimers (called A/B dimers) where the FG-loop of the B monomer adopts a different conformation from that in the A monomer. In the remaining 30 dimers (C/C dimers) the FG-loops of both adopt the same conformation^13^ (Fig. 1a). Binding of RNA stem-loops from within the viral genome can trigger the conformational change from the symmetric to the asymmetric dimer confirmation via a displacement of the kinetic energy favoring conversion from the C/C to the A/B conformer^14,15^ Multiple dispersed RNA stem-loops in the genome with a shared sequence motif, termed packaging signals, cooperatively ensure efficient virus assembly^16–22^. The differences in FG-loop conformation are a determinant of the local symmetry of the MS2 capsid shell. A/B dimers are located in groups of five around the particle 5-fold axes and are responsible for pentamer formation. This is a consequence of the fact that in the B monomer the FG-loop is flipped (away from the cage lumen) which allows closer packing of coat proteins around the 5-fold axes, enabling the 5-fold symmetry arrangement of five B subunits. In the alternative monomer conformation, the FG-loop accommodates a rigid β hairpin-like structure and allows packing only in groups of six dimers around the particle 3-fold axes. C/C dimers are symmetrical, with both FG-loops in hairpin conformation, and participate in neighboring 6-fold clusters, thus acting as a staple between them. According to Euler’s theorem, exactly 12 five-fold clusters are needed to build a closed shell, and therefore for the protein capsids we observe, both types of dimers are required to form a closed protein capsid.

MS2 VLPs have been used for a number of applications, including encapsulation of heterologous nucleic acids to serve as RNA “armors” in preparation of internal controls for qPCR^23–25^; as delivery vehicles^1,3,26^; for incorporation of immunogenic epitopes displayed on the particle surface^12,27–31^; and development of the so-called MS2-tagging syste^m32,33^, which allows the study of RNA-protein interactions in cells. Recently MS2 VLPs carrying fragments of the SARS-CoV-2 genome have been developed as control standards for diagnostic tests^34^.

MS2 itself has been engineered to assemble particles of different T-number in the presence of RNA cargo: A single mutation (S37P) is able to convert the VLP from a *T* = 3 wild-type particle of 27 nm in diameter to a *T* = 1 particle of only 17 nm in diameter^35^. Furthermore, recent work has shown that the wildtype capsid also forms *T* = 4 structures which account for about 6% of the total particles formed^36^.

Here we have shown how multiple different geometries resulting in larger size can be achieved via modifications to the MS2 capsid protein which also increases the yield of “unnatural” larger capsids until they are the largest fraction or otherwise are enriched multi-fold compared to their natural occurrence. Specifically, we show that inserting amino acid sequences into an externally facing loop of one monomer of the MS2 capsid dimer not only provides sites for attachment of arbitrary proteins of interest but, through its effect on the FG-loop conformation, alters the pentamer/hexamer equilibrium leading to a shift towards larger capsids being formed (Fig. 1b). Moreover, we explain systematically, using a kinetic model, how different MS2 VLP geometries form (Fig. 1c, d) and how changes engineered in the capsid protein structures impact the abundance of the “wild type” form of the MS2 capsid and its variants (Fig. 1e).

## Results

### Design and production of modified MS2 VLPs

**Fig. 1:**
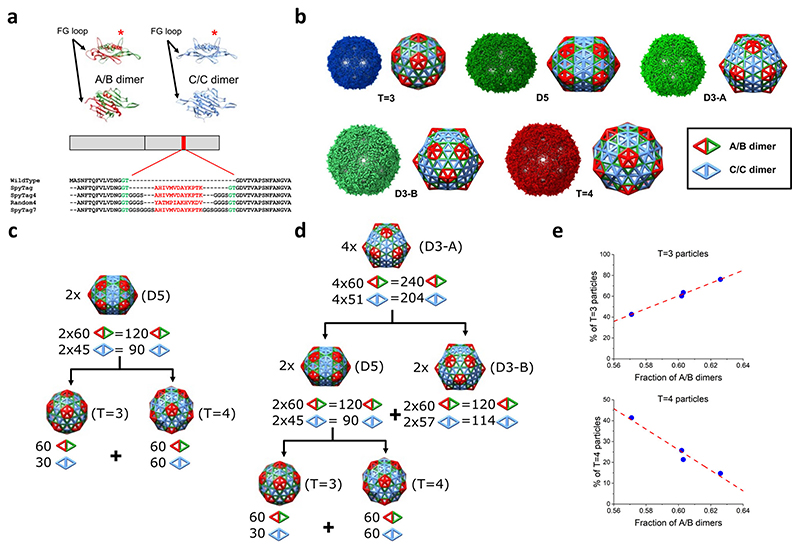
Virus-like particles derived from bacteriophage MS2. **a** Orthogonal views of the A/B (top left) and C/C (top right) dimers. The FG-loop determining dimer conformation is indicated by arrows, and the insertion site by a red asterisk. The MS2 coat protein dimer constructs, with the insert highlighted in red, is shown underneath, together with a sequence alignment to the wild-type protein (black), with the restriction site-dependent amino acids in green and additional amino acids in bold black, respectively. **b** The reconstructed cryo-EM densities of all identified MS2 VLP variants, with schematic representation of A/B and C/C dimer tilings (left and right, respectively); the symmetry of each structure is indicated below. **c**, **d** Examples of equivalent particle stoichiometries: **c** two D5 particles can be converted into one *T* = 3 and one *T* = 4 particles; and d four D3-A particles can be converted into two D3-B and two D5 particles, which can be subsequently converted into one *T* = 3 and one *T* = 4 particles. **e** The percentages of *T* = 3 and *T* = 4 particles in the ensemble depend linearly on the fraction of asymmetric dimers; data points corresponding to, from left to right, SpyTag4; Random4; SpyTag7; and SpyTag, reveal a linear trend (dotted line) with respect to the fraction of A/B dimers.

We designed MS2 VLPs to provide an externally displayed anchor for attachment of proteins/peptides of choice while simultaneously investigating effects on VLP morphology. To achieve this, we utilized the Spytag—Spycatcher system^37^. This consists of a 13-residue peptide (called Spytag) and a 116-residue complementary domain (called Spycatcher). When the two are mixed, the holo Spy protein is spontaneously reconstituted. This technology has previously been used to create VLPs displaying immune epitopes or cellular receptor ligands for further development of vaccines or delivery vehicles, respectively^38^. We designed MS2 CP constructs as tandem dimer fusions, which is known to confer tolerance to peptide insertions in one monomer without interfering with particle assembly^39^. We inserted the Spytag sequence at the *KpnI* (144 G) position (Fig. 1a) as has been used for His-tag insertion^40^. In order to optimize the Spytag exposure at the particle surface (and thus confer the best availability for the Spycatcher attachment) we also created variants with longer flanking linkers. The designed constructs were generated by cloning (all primer sequences are listed in Supplementary Table 1) and verified by sequencing. Proteins were expressed in *E. coli* and the resulting VLPs were extracted and purified as described in Methods. These variants of MS2 VLPs were named “SpyTag”, “SpyTag4”, “Spy-Tag7”, and “Random4”, reflecting the characteristics of the insert sequence as further described below.

### Characterization of MS2 VLPs

**Fig. 2:**
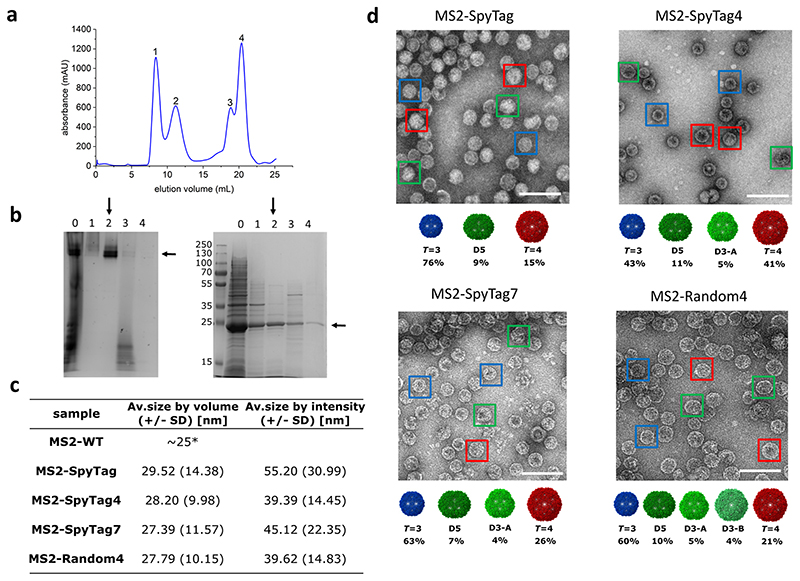
MS2 VLPs purification and analysis. **a** A representative size exclusion purification profile for an MS2 VLP; **b** native PAGE (left) and SDS-PAGE (right) analysis of eluted fractions: lane numbers correspond to peak numbers on the chromatogram; “0” denotes sample before SEC. Vertical arrows indicate fractions containing purified VLP and are used for further analysis. Horizontal arrows indicate bands corresponding to the expected molecular weight (of the coat protein dimer in SDS-PAGE and the VLP in native PAGE); **c** Table showing representative DLS measurements of hydrodynamic diameter of the MS2-SpyTag particles present in fractions collected from peak 2 (on the chromatogram). Volume distribution (top left) in comparison with the intensity distribution (top right); together with diameter measurements for the entire ensemble of MS2 samples, *adapted from ref. ^53^ **d** Transmission electron microscopy images of MS2 VLP variants. Color frames show different morphologies of the assembled VLPs: *T* = 3, *T* = 4 and the lower symmetry variants (D5, D3-A, and D3-B) in blue, red, and green, respectively with the overall percentage of each morphology identified in the analyzed samples; scale bar:100 nm.

Purified MS2 VLPs were initially characterized using size exclusion chromatography (SEC), native PAGE, and SDS-PAGE. For all VLP variants (MS2-SpyTag, MS2-SpyTag4, MS2-SpyTag7, and MS2-Random4) four peaks were typically present in SEC profiles (Fig. 2a): peak 2 corresponds to properly assembled particles in the MDa range as confirmed by native PAGE (Fig. 2b). The other peaks (1, 3, and 4) are present at different levels depending on the variant (not shown) and likely consist of unassembled or partially assembled MS2 CP (~29 kDa, see SDS-PAGE, Fig. 2b).

All variants of purified VLPs, collected from peak 2 fractions, were analyzed using DLS. We noted that for particles carrying longer insertions (MS2-SpyTag4, MS2-SpyTag7, and MS2-Random4) the mean hydrodynamic diameter was smaller than for the SpyTag variant (Fig. 2c). Moreover, when size distributions are analyzed by intensity, the shape of the peaks indicates the possibility of more than one population of particles with similar but not identical hydrodynamic radii (Fig. 2c). Further investigation using negative stain TEM confirmed the expected VLP structure for all produced proteins and also confirmed differences in diameter depending on the presence and length of the linker (Fig. 2d), with diameter magnitudes observed in the order MS2-SpyTag4 ≈ MS2-Random4 > MS2-SpyTag7 > MS2-SpyTag.

### Attachment of external proteins

**Fig. 3:**
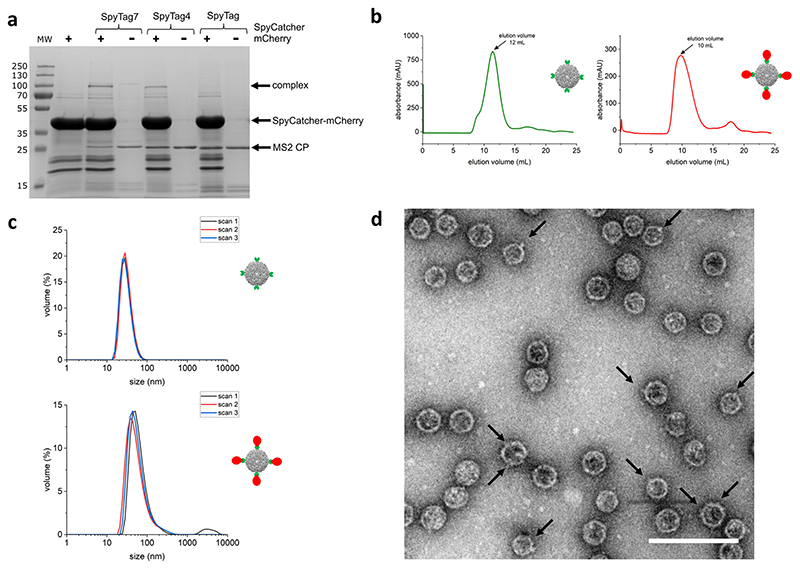
MS2 VLP decoration with the Spy Catcher – mCherry protein. **a** SDS-PAGE gel showing the interaction of the SpyCatcher–mCherry protein with different versions of the MS2 coat protein fused with SpyTag variants; **b** Size exclusion elution profiles of naked MS2 VLPs (MS2-SpyTag4 left/green) and the same VLPs decorated with SpyCatcher–mCherry (right/red). In both cases, samples were pre-purified using an ultrafiltration device with MW cut off 100 kDa. **c** DLS profiles (three independent scans of the same sample) of the SpyTag modified MS2 VLPs alone (top) and after addition of SpyCatcher–mCherry partner (bottom); **d** TEM image of SpyCatcher–mCherry decorated MS2 VLPs, with black arrows pointing to the SpyCatcher–mCherry protein on the surface of MS2 VLPs (scale bar: 100 nm).

In order to confirm the presence of Spytag at the expected location, being both external and accessible, we attached the SpyCatcher–mCherry fusion protein to the assembled VLP. MS2-Spytag VLP variants (MS2-SpyTag, MS2-SpyTag4, MS2-SpyTag7) were incubated with SpyCatcher–mCherry protein and then analyzed by SDS-PAGE (Fig. 3a). Bands corresponding to CP dimers conjugated to the SpyCatcher–mCherry protein can be seen in the case of VLP variants where Spytag is flanked by linkers (MS2-SpyTag4, MS2-SpyTag7), but not in the case of MS2-SpyTag. As a next step, reaction mixtures containing both protein moieties were pre-purified using an ultrafiltration device and separated using size exclusion chromatography, allowing removal of the free SpyCatcher–mCherry protein and providing additional confirmation that MS2 particles are efficiently decorated (Fig. 3b). Finally, purified MS2 VLPs decorated with SpyCatcher–mCherry protein were subjected to DLS measurement (Fig. 3c) and inspected using transmission electron microscopy (TEM, Fig. 3d) with results demonstrating the integrity of the particles as well as an increase in their volumes.

### Cryo-EM structures of modified MS2 VLPs.

All MS2 VLP variants underwent full cryo-EM analysis (as described in Methods (Supplementary Figs. 1–4)). In total, 16 structures were identified and determined: three for MS2-SpyTag (Supplementary Figs. 1 and 6) with *T* = 3, D5 and *T* = 4 symmetries, four for MS2-SpyTag4 (Supplementary Figs. 2 and 7) and MS2-SpyTag7 (Supplementary Figs. 3 and 8) with *T* = 3, D5, D3 (D3-A) and *T* = 4 symmetries, and five for MS2-Random4 (Supplementary Figs. 4 and 9) with *T* = 3, D5, D3 (D3-A and D3-B) and *T* = 4 symmetries. All structures were determined via analyses starting with C1 symmetry with higher-order symmetries gradually imposed in order to find the highest possible symmetry consistent with the data. The different symmetries imply that there are different ratios between the numbers of local 5- and 6-fold clusters in the corresponding particle morphologies. As the B monomers form the 5-fold clusters and the A and C monomers the 6-fold clusters in the MS2 capsid, this suggests that the relative ratios between A/B and C/C dimers must be different and that this ratio can be used to control particle morphology. All cryo-EM reconstructions were deposited in EMDB—see Supplementary Table 2.

Since the monomers in MS2 dimers either partake in a 5-fold cluster (in the case of the B monomer) or in a 6-fold cluster (in the case of the A and C monomers), any capsid geometry assembled from such building blocks must be geometrically identical to fullerene structures, with fullerene edges mapping onto the short diagonals of the MS2 coat proteins dimers. Analysis of the structures obtained in the experiment showed that in cases where there is more than one possible fullerene isomer, MS2 particles accommodate geometries related to the most energetically stable one (Supplementary Fig. 5) and with the highest possible symmetry.

### Geometrical interpretation of the results

The five distinct types of particles observed must each have 12 pentagonal clusters according to Euler’s theorem, and therefore precisely 60 A/B dimers. The numbers of C/C dimers, on the other hand, differ for distinct particle types (Supplementary Table 3). The ensemble of particles observed for each SpyTag variant therefore also differs in its cumulative A/B:C/C dimer content (Supplementary Table 4). However, the relative ratio of A/B:C/C dimers is not sufficient to explain the observed particle distribution, because the same ratio could also be used, from a purely stoichiometric perspective, to construct different combinations of particles. This is because the numbers of A/B and C/C dimers in two D5 particles are the same as for a *T* = 3 plus a *T* = 4 particle, and similarly, two D3-A particles are equivalent to a D5 plus a D3-B particle (Fig. 1c, d). For each SpyTag variant, we computed all possible ensembles consistent with the observed numbers of A/B and C/C dimers (Supplementary Table 4), demonstrating that the scenarios seen in the experiment do not favor the higher symmetry particles (*T* = 3 plus *T* = 4) over D5 particles. A similar phenomenon has previously been seen^41^ where particles with *T* = 4 and D5 symmetry have been observed that have comparable energy and are both minimum energy structures. Similarly, we also observe here particles with different symmetries that could be minimum energy structures. In order to determine their relative numbers, we use an assembly kinetics approach.

### Assembly kinetics reveals the origin of distinct particle morphologies

We constructed an assembly model based on reaction kinetics, that includes the interconversion between the C/C and A/B dimers, as well as the association of dimers according to a tree that indicates bifurcations in the assembly pathways resulting in the observed particle geometries (Supplementary Fig. 10). Under otherwise identical conditions, different interconversion rates between C/C and A/B dimers result in the distinct particle morphologies observed for SpyTag, Random4, and SpyTag7 (Supplementary Table 5) suggesting that conversion from the C/C to the A/B conformer is indeed differentially affected by the Spytag insertions. SpyTag4 is the most affected with the lowest conversion rate, consistent with presenting the highest yield of *T* = 4 particles for any of the cases. The rate consistent with obtaining mostly native *T* = 3 particles is significantly higher than those obtained here, implying that Spytag insertions indeed have a major impact on the conversion from C/C to A/B compared with wild-type virus (Supplementary Table 6). Interestingly, the length of the SpyTag4 and Random4 inserts is identical, suggesting that insert size alone is not a predictor of the experimental outcome. We note that the elastic energy can influence the structure of the assembly products. Whilst such contributions are implicitly contained in the parameters of the assembly model, which are inferred from the experimental data, it is important to assess the contributions that elastic properties make to the particle breakdown. In particular, the total energy of an empty capsid has an elastic contribution from bending and stretching, and the energy barrier between different particle morphologies increases with the elastic energy per subunit^42^ (see Methods).

## Discussion

In this work, we have discovered that insertion of particular amino acid sequences at a certain position in the CP protein, results in a significant shift towards larger VLP sizes. The abundance of such larger particles depends on the amino acid sequence meaning that a level of selective control is possible. Experimental validation was achieved by determining 16 cryo-EM structures from the designed protein variants, five of which are unique in terms of their overall geometry. We have been able to identify and confirm a previously reported^43^ MS2-like capsid with *T* = 4 geometry. In addition, we were able to solve structures of three previously unreported MS2 capsid variants: one with D5, and two with D3 symmetry (D3-A and D3-B). The structures showed no evidence of significant nucleic acid cargo encapsulated within the VLPs, ruling out a cargo effect on VLP size. Similarly, VLPs were produced in identical buffer conditions meaning that solvent and salt effects were not responsible. We note, however, that nucleic acid cargoes, to be delivered via such vectors in applications, could impact on container geometry. In particular, any cargo containing multiple packaging signals would likely impact the ratio of A/B and C/C dimers and thus bias container geometry to morphologies with fewer C/C dimers. It is also expected that cargo length impacts capsid geometry^44^.

In order to understand how the insertion of additional amino acid sequences in an external loop triggers this effect, we developed a kinetic model of particle assembly. The model suggests that the difference in experimental outcome for distinct Spytag inserts is due to a delay in conversion from C/C to A/B, which could be mediated by a change in the dynamic behavior of the protein dimer as a result of the insertions. This would be consistent with our understanding that the dynamic properties of the C/C dimer affect the conversion to A/B, and that this shift can be triggered by binding to an RNA packaging signal^11^. However, whilst packaging signals promote this shift, Spytag insertions appear to disfavor it. As a notable example, the largest difference between ideal and actual scenarios (Supplementary Table 8) is seen for SpyTag and SpyTag4. Our model suggests that this is because they are slower in converting C/C into A/B dimers than SpyTag7 and Random4. Note that they both share the same AHIVMVDAYKPTK insertion, with the flanking GGGS in Spy-Tag4, apparently accounting for the slower conversion. In contrast, the longer flanks of SpyTag7 and randomization of the inserted region have a less marked effect. The exact mechanism accounting for this is not clear. However, the SpyTag sequence is structured, forming a beta hairpin and it is possible that this increased rigidity stabilizes the C/C dimer slowing conversion to the A/B form. A random sequence (Random4) being unstructured has reduced stabilizing effect as does SpyTag7 which has a much greater proportion of unstructured linker sequence. In contrast, SpyTag, having no linker may be prevented from forming a stable structure. In agreement with experimental results, our analysis suggests that the production of desired particle morphologies can be achieved via the choice of SpyTag insert, affording unprecedented control over the production of the desired particle morphologies.

## Conclusion

In this work, we have shown that a VLP can be modified such that its diameters are significantly shifted towards larger sizes with altered symmetries. Given the proven and potential applications of MS2^1–3^ and other VLPs in virus nanotechnology, the ability to produce containers of larger volume with enhanced carrying capacity is highly desirable. Further, understanding how the inserts alter assembly kinetics and the resulting VLP morphology gives a deeper control of VLP assembly overall. Taken together, these results are the first essential step towards production of bespoke VLPs with desired morphologies and assembly/disassembly properties.

## Methods

### Cloning, expression, and purification of MS2 VLPs.

The MS coat protein (CP) gene was designed as a tandem dimer, with the second part of the dimer bearing a KpnI restriction site allowing for foreign insertions, as reported previously^39,40^. A synthetic gene coding for MS2 CP dimer with Spytag insertion (Supplementary Methods) cloned into pET28a was purchased from BioCat GmbH (Germany). Variants with longer linkers flanking the Spytag insertion and the random peptide insertion were generated by PCR using appropriate primers (Supplementary Table 1) and then subcloned to pET28a harboring the MS2 CP dimer gene, using the KpnI restriction site.

For expression, *E. coli* BL21(DE3) cells were transformed with appropriate plasmids and grown with shaking at 37 °C until OD_600_ = 0.6, induced with 1 M IPTG and then further shaken at 18 °C for 16 h. Cells were harvested by centrifugation, resuspended in 50 mM Tris-HCl, pH 7.9, 50 mM NaCl, 5 mM MgCl_2_, 5 mM CaCl_2_ and lysed by sonication at 4 °C in the presence of protease inhibitors (Thermo Scientific). Lysates were clarified by centrifugation and Viscolase (10,000 U/1 L culture; AA Biotechnology, Poland) was added to supernatant fraction, incubated for 20 min at 37 °C, followed by 10 min incubation at 50 °C. The supernatant fraction was then centrifuged again and mixed 1:1 (V/V) with 3.7 M (NH_4_)_2_SO_4_, and the reaction was incubated overnight at 4 °C. Precipitated proteins were harvested by centrifugation for 10 min, 11,000 × g, at 4 °C and resuspended in PBS. The solution was then filtered through 0.2 μm membrane filters (VWR) and passed through an Amicon MWCO 100 kDa (Millipore) filtering device, in order to remove residual (NH_4_)_2_SO_4_ and low molecular mass proteins. Protein concentration was adjusted to 2.5–5mgmL^−1^, as measured by Nanodrop (A_280_) and SEC—purified in PBS buffer, using Superose 6 Increase column (GE Healthcare) connected to an AKTA FPLC system.

### SDS-PAGE and native PAGE

MS2 VLPs variants were analyzed by electrophoresis in both denaturing and native conditions. For SDS-PAGE, samples were separated on 12% gels Tris/Glycine gels using standard Laemmli protocol, whereas for non-denaturing electrophoresis Bis-Tris gels 3-12% gradient gels were used (Life Technologies), following the manufacturer’s recommendations. A Chemidoc detector (BioRad) was used for fluorescence detection with excitation at 546 nm. Gels were stained in InstantBlue (Expedeon).

### Dynamic light scattering

Dynamic light scattering (DLS) was carried out using a Zetasizer Nano ZS (Malvern). Samples of purified MS2 VLPs were diluted to 0.05 mg mL^-1^ (A_280_), 12045 × g centrifuged for 5 min, and transferred to plastic/quartz cuvette (ZEN 2112). Measurements were performed in triplicates (15 runs for each measurement). Only measurements meeting Malvern software quality criteria were used for analysis.

### Transmission electron microscopy

Samples of purified MS2 VLPs were diluted to 0.05 mg/mL, centrifuged at maximum speed for 15 min, and additionally filtered through 0.1 μm membrane filters (VWR). Samples were then applied onto hydrophilized carbon-coated copper grids (STEM Co.), negatively stained with 1% uranyl acetate, and visualized using a JEOL JEM-1230 transmission electron microscope (TEM) at 80 kV.

### SpyCatcher–mCherry production and interaction with MS2 VLP

The His-tagged SpyCatcher–mCherry construct was created by PCR amplification of the mCherry gene from a pACYC Duet plasmid (a kind gift from Yusuke Azuma) and its sub-cloning to pET28a harboring His-tagged Spycatcher fragment (synthetic construct, Biocat, Germany). The final construct was verified by sequencing.

*E. coli* BL21(DE3) cells were transformed with the above plasmid and protein expression and extraction were conducted as described above for MS2 CP. The protein was purified using Ni-NTA and following standard purification protocol. Briefly, the cell extract was incubated with agarose beads coupled with Ni^2+^-bound nitrilotriacetic acid (His-Pur Ni-NTA, Thermo Fisher Scientific) pre-equilibrated in 50 mM Tris, pH 7.9, 150 mM NaCl, 20 mM imidazole (Buffer A). After three washes of the resin (with Buffer A) the protein was eluted with 50 mM Tris, pH 7.9, 150 mM NaCl, 300 mM imidazole (Buffer B). Fractions containing protein of interest were pooled and passed through Sephadex 25 (Millipore) columns in order to remove imidazole. Final protein concentration was measured by Nanodrop at 280 nm wavelength.

Attachment of purified SpyCatcher–mCherry to MS2 VLP was conducted by mixing the two in a range of different molar ratios, followed by 90 min incubation at room temperature. The interaction efficiency was evaluated by SDS-PAGE whereas efficient particle decoration was confirmed using size exclusion chromatography (as described above), preceded by filtration on the Amicon column with MW cut off 100 kDa (Merck). The optimized ratio used in the presented results (Fig. 3b) was 4: 1 (SpyCatcher–mCherry: MS2 VLP).

### Cryo-electron microscopy

Purified samples of MS2 VLPs at ~1 mg mL^−1^ concentration were flash-frozen in liquid ethane using an FEI Vitrobot (sample volume 4 μL, blot force 0, blot time 4 s) on previously glow-discharged copper grids (Quantifoil, Cu 1.2/1.3, mesh 400). All grids were imaged with a 300 kV acceleration voltage using a Titan Krios microscope armed with a Gatan K3 camera (0.86 A/px, 40 frames movies). Raw micrographs were motion corrected using WARP^45^ with all further steps carried out using the CryoSPARC v2.15.0 software package^46^. CTF values were calculated in patch mode using Patch CTF. Micrographs were accepted for particle picking when meeting a criterion of CTF fit better than 8 Å (CTF ≤ Å). All reported resolution values are a result of independent half maps analysis with gold-standard FSC criterion (FSC = 0.143). All figures containing cryo-EM maps were prepared using either UCSF Chimera^47^ or ChimeraX^48^.

### MS2 VLP assembly model

Particle assembly is modeled via reaction kinetics, that encodes the interconversion between C/C and A/B dimers with forward rate *f* and backward rate *b* (Eq. 1):

(1) $$\text{C}/\text{C}\underset{b}{\overset{f}{⇌}}\text{A}/\text{B}$$

Assembly starts with formation of particle 5-fold axes according to the following reactions (Eqs. 2 and 3):

(2) $$i\text{A}/\text{B}+\text{A}/\text{B}\underset{b_{1}}{\overset{f_{1}}{⇌}}(i+1)\text{A}/\text{B},1\leq i\leq 3,$$

(3) $$4\text{A}/\text{B}+\text{A}/\text{B}\underset{b_{2}}{\overset{f_{2}}{⇌}}5\text{A}/\text{B},$$

where $\frac{b_{1}}{f_{1}}=e^{\frac{\text{Δ}G}{K_{B}T}}$ and *K_B_* is the Boltzmann constant, *T* is temperature, and ΔG is the binding free energy which is −2.7 kcal *M* ^−149^. For the last reaction, the binding free energy is −5.4kcal *M* ^−1^ as there are two binding sites for the fifth A/B. This is followed by the acquisition of five C/C dimers around the 5 A/B complex (Eq. 4):

(4) $$5\text{A}/\text{B}:i\text{C}/\text{C}+\text{C}/\text{C}\underset{b_{2}}{\overset{f_{2}}{⇌}}5\text{A}/\text{B}:(i+1)\text{C}/\text{C }0\leq i\leq 4.$$

As this early assembly intermediate is shared by all particles, we assume that the first branching of the assembly pathways, resulting in the observed particle geometries, occurs at this point (cf. *split 1* in Supplementary Fig. 10a, b). At this stage, we assume that A/B and C/C dimers bind with rates $f_{\text{elong }}^{ab}$ and $f_{\text{elong }}^{\text{cc}}$ , respectively (*f*_1_ = 10^3^M^−1^S^−1^, $f_{2}=f_{\text{elong }}^{\text{ab}}=f_{\text{elong }}^{\text{cc}}$ = 10^6^ M^−1^S^−1^ ^50, 51^), to the 5 A/B:5 C/C intermediate. These additions are based on a tree that indicates bifurcations in the assembly pathways whenever the addition of an A/B or C/C dimer commits the intermediate to the assembly of a distinct particle type (Supplementary Fig. 10a). To move towards the formation of *T* = 3 particles, the intermediate 5 A/B + 5 C/C must bind to an A/B dimer, whilst recruitment of a C/C dimer will result in the formation of *T* = 4 particles (Supplementary Fig. 10b). This has been modeled as follows (Eqs. 5 and 6):

(5) $$5\text{A}/\text{B}:5\text{C}/\text{C}+\text{A}/\text{B}\underset{b_{2}}{\overset{f_{\text{elong }}^{\text{ab}}}{⇌}}6\text{A}/\text{B}:5\text{C}/\text{C},$$

(6) $$5\text{A}/\text{B}:5\text{C}/\text{C}+\text{C}/\text{C}\overset{(split\;1)\times f_{\text{elong }}^{\text{cc }}}{\underset{b_{2}}{⇌}}5\text{A}/\text{B}:6\text{C}/\text{C}.$$

We assume that the forward rate of the second reaction is reduced by the factor *split 1* (Supplementary Table 5) to reflect the fact that it is a bifurcation from the wild type (*T*=3) pathway. Supplementary Fig. 10b shows that in the assembly pathway of *T* = 3 (*T* = 4) particles, the intermediates 15 A/B:8 C/C (15 A/B:11 C/C) must acquire an A/B dimer to continue towards a *T* = 3 (*T* = 4) particle geometry. However, if they acquire a C/C dimer, they will continue towards the formation of D3 particles (cf. Supplementary Fig. 10b). Thus, in the model, we assume that 15 A/B:8 C/C (15 A/B:11 C/C) can bifurcate towards the formation of D3 particles by binding to a C/C dimer. Similarly, the rate of this split is reduced by the factor *split 2* (*split 3*) as D3 particles have a lower symmetry compared with *T* = 3 (*T* = 4) particles, and we model these splits as for *split 1*. The assembly pathways of *T* = 3 and D5 particles are similar until 30 A/B:20 C/C (Supplementary Fig. 10c), where recruitment of an A/B (C/C) dimer biases particle formation towards a *T* = 3 (D5) particle type (cf. *split 4* in Supplementary Fig. 10a). Supplementary Fig. 10d illustrates that the assembly pathways of D3-A and D3-B particles are similar until 44 A/B:27 C/C, where recruitment of A/B dimer results in the formation of D3-A particles, and that of C/C dimers in the formation of D3-B particles (cf. *split 5* in Supplementary Fig. 10a, d).

In the absence of a split in the assembly tree, the transition from assembly intermediate *n*_1_A/B : *m*_1_C/C to *n*_2_ A/B : *m*_2_C/C is modeled as the random binding of (*n*_2_ − *n*_1_)A/B and (*m*_2_ − *m*_1_)C/C dimers according to the following matrix, modeling the successive recruitment of individual A/B and C/C dimers in an *n*_2_ A/B : *m*_2_ C/C array:

(7) $$\left(\begin{array}{ccccccc} n_{1}\text{A}/\text{B}:m_{1}\text{C}/\text{C} & \underset{\to }{f_{\text{elong}}^{cc}} & n_{1}\text{A}/\text{B}:\left(m_{1}+1\right)\text{C}/\text{C} & \underset{\to }{f_{\text{elong}}^{cc}} & \ldots & \underset{\to }{f_{\text{elong}}^{cc}} & n_{1}\text{A}/\text{B}:m_{2}\text{C}/\text{C}\\ f_{\text{elong }}^{\text{ab}}↓ & & f_{\text{elong }}^{\text{ab}}↓ & & \ldots & & f_{\text{elong }}^{\text{ab}}↓\\ \left(n_{1}+1\right)\text{A}/\text{B}:m_{1}\text{C}/\text{C} & \underset{\to }{f_{\text{elong}}^{cc}} & \left(n_{1}+1\right)\text{A}/\text{B}:\left(m_{1}+1\right)\text{C}/\text{C} & \underset{\to }{f_{\text{elong}}^{cc}} & \ldots & \underset{\to }{f_{\text{elong}}^{cc}} & \left(n_{1}+1\right)\text{A}/\text{B}:m_{2}\text{C}/\text{C}\\ f_{\text{elong }}^{\text{ab}}↓ & & f_{\text{elong }}^{\text{ab}}↓ & & \ldots & & f_{\text{elong }}^{\text{ab}}↓\\ \vdots & & \vdots & & \vdots & & \vdots \\ n_{2}\text{A}/\text{B}:m_{1}\text{C}/\text{C} & \underset{\to }{f_{\text{elong}}^{cc}} & n_{2}\text{A}/\text{B}:\left(m_{1}+1\right)\text{C}/\text{C} & \underset{\to }{f_{\text{elong}}^{cc}} & \ldots & \underset{\to }{f_{\text{elong}}^{cc}} & n_{2}\text{A}/\text{B}:m_{2}\text{C}/\text{C} \end{array}\right).$$

These kinetic equations are the basis of stochastic simulations performed with the Gillespie algorithm^52^ implemented in Fortran.

### Parameter values

We note that our model depends on five parameters, one for each split in the assembly tree, that identify the likelihood that assembly occurs along a different branch (*splits* rates). These parameters have been fitted with respect to data for one scenario, and then kept the same for the other scenarios in order to make the results comparable (Supplementary Table 5). The default rate for the *splits* is first chosen for all but the SpyTag4 scenario, as the latter leads to a much higher yield in *T* = 4 particles compared to the others. *Splits* rates are chosen to reflect the symmetry of the particles, as there are more equivalent contact points for particles with higher symmetry. Consistent with this, *split 2* is the lowest, as it leads to the formation of D3 particles with the lowest symmetry on the *T* = 3 (wild type) branch. *Split 3* is slightly larger, although it leads to D3 particles, as it occurs on the *T* = 4 branch of the assembly tree. *Split 1* is the largest as it occurs at the start of the assembly process and can lead to *T* = 4 particles whose assembly intermediates offer the largest number of symmetry-equivalent positions for incoming subunits. *Split 4* is smaller than *split 1*, as D5 is of lower symmetry than *T* = 4. *Split 5* is slightly smaller than *split 1* because it occurs at the end of the assembly process, and since D3-A contains fewer C/C dimers than D3-B^52^. At that point, only the conversion rate from the symmetric C/C to the asymmetric A/B dimer (*f*) remains a free parameter in the model, and it is identified for each scenario based on the experimentally observed outcomes in relative particles numbers (Supplementary Table 7). For the case of SpyTag4, for which the level of *T* = 4 particles is much higher than for the other cases, variation of *f* alone is not sufficient to account for the data. We note that the best fit is obtained when *f* is smaller than in all other cases, implying that there is a resistance of C/C dimers to convert into A/B in this case. This is likely due to the dynamic properties of the dimer as a result of the SpyTag4 insert and may also affect the C/C binding rate to the assembly intermediates. We reflect this by reducing the value of the elongation rate of C/C dimers ( $f_{\text{elong }}^{\text{cc}}$ ). This also implies that C/C dimers are more likely to occupy positions that require less dynamic flexibility, i.e., positions with lower curvature where C/C dimers need to bend less in order to attach. Consistent with this, C/C recruitment is higher at *split 1* and *split 4* (Supplementary Table 5) as they lead to particles with lower curvatures, as *T* = 4 is larger than *T* = 3, and D5 has a cylindrical shape.

### Elastic properties of different particle morphologies

The elastic energy per subunit for each particle type has been determined with reference to the tiling by counting dimers in equivalent positions, i.e., in groups with comparable stretching and bending, for each structure. We assume that the elastic energy for each A/B and C/C dimer in a *T* = 3 particle is $\varepsilon_{0}^{T=3}$ and $\varepsilon_{1}^{T=3}$ , respectively, and $\varepsilon_{1}^{T=4}$ and $\varepsilon_{1}^{T=4}$ for a *T* = 4 particle. In D5, D3-A, and D3-B particles there are C/C dimers that are bounded by only C/C dimers (Supplementary Fig. 10c, d). As we do not have a dimer in *T* = 3 and *T* = 4 particles with this behavior, we introduce the additional elastic energy ε_2_. The elastic energy per subunit (i.e. per dimer) is thus:

$$\begin{array}{l} E_{T=3}=\frac{2}{3}\varepsilon_{0}^{T=3}+\frac{1}{3}\varepsilon_{1}^{T=3},\\ E_{T=4}=\frac{1}{2}\varepsilon_{0}^{T=4}+\frac{1}{2}\varepsilon_{1}^{T=4},\\ E_{D5}=\frac{2}{7}\left(\varepsilon_{0}^{T=3}+\varepsilon_{0}^{T=4}\right)+\frac{4}{21}\left(\varepsilon_{1}^{T=3}+\varepsilon_{1}^{T=4}\right)+\frac{1}{21}\varepsilon_{2},\\ E_{D3-A}=\frac{10}{37}\left(\varepsilon_{0}^{T=3}+\varepsilon_{0}^{T=4}\right)+\frac{5}{37}\varepsilon_{1}^{T=3}+\frac{10}{37}\varepsilon_{1}^{T=4}+\frac{2}{37}\varepsilon_{2},\\ E_{D3-B}=\frac{8}{39}\varepsilon_{0}^{T=3}+\frac{4}{13}\varepsilon_{0}^{T=4}+\frac{1}{13}\varepsilon_{1}^{T=3}+\frac{14}{39}\varepsilon_{1}^{T=4}+\frac{2}{39}\varepsilon_{2}. \end{array}$$

As *T* = 3 and *T* = 4 particles are similar, for simplicity we assume that in a good approximation $\varepsilon_{0}^{T=3}=\varepsilon_{0}^{T=4}=\varepsilon_{0}$ and $\varepsilon_{1}^{T=3}=\varepsilon_{1}^{T=4}=\varepsilon_{1}$ . Introducing dimensionless parameters $k_{1}=\frac{\varepsilon_{1}}{\varepsilon_{0}}$ and $k_{2}=\frac{\varepsilon_{2}}{\varepsilon_{0}}$ then reduces these equations to

$$\begin{array}{l} E_{T=3}/\varepsilon_{0}=\frac{2}{3}+\frac{1}{3}k_{1},\\ E_{T=4}/\varepsilon_{0}=\frac{1}{2}+\frac{1}{2}k_{1},\\ E_{D5}/\varepsilon_{0}=\frac{4}{7}+\frac{8}{21}k_{1}+\frac{1}{21}k_{2},\\ E_{D3-A}/\varepsilon_{0}=\frac{20}{37}+\frac{15}{37}k_{1}+\frac{2}{37}k_{2},\\ E_{D3-B}/\varepsilon_{0}=\frac{20}{39}+\frac{17}{39}k_{1}+\frac{2}{39}k_{2}. \end{array}$$

These define different areas in parameter space given the relative stretching and properties of C/C dimers in different positions. The red region in Supplementary Fig. 11 indicates the area for which *E_T_*_=3_ < *E_T_*_=4_ < *E_D_*_5_ < *E_D_*_3−A_ < *E_D_*_3−*B*_. It is worth noticing that in this area the difference between *k*_2_ and *k*_1_ is bigger than the difference between *k*_1_ and 1, i.e. the jump in the level of elastic energy from ε_1_ to ε_2_ is bigger than the jump from ε_0_ to ε_1_. This is consistent with the fact that the C/C dimer that is only bound to other C/C dimers (ε_2_) is in a flatter position. It also reflects the order in particle numbers seen in the experiment, *T* = 3 > *T* = 4 > D5 > D3-A > D3-B (Supplementary Table 7). This demonstrates that elastic properties are important for the assembly outcome and can account for the rank order of the particle types. However, in order to determine the precise values and differences for each SpyTag option, we refer to the kinetic model above.

## Supplementary Material

Supplementary information The online version contains supplementary material available at https://doi.org/10.1038/s43246-022-00229-3.

Supplementary Materials

## Data Availability

The data that support the findings of this study are available from the corresponding author on reasonable request. The cryo-EM density maps have been deposited in the Electron Microscopy Data Bank under accession codes: EMD-12778 (MS2-SpyTag, *T* = 3), EMD-12779 (MS2-SpyTag, *T* = 4), EMD-12780 (MS2-SpyTag, D5), EMD-12781 (MS2-SpyTag4, *T* = 3), EMD-12782 (MS2-SpyTag4, *T* = 4), EMD-12783 (MS2-SpyTag4, D5), EMD-12784 (MS2-SpyTag4, D3-A), EMD-12785 (MS2-Random4, *T* = 3), EMD-12786 (MS2-Random4, *T* = 4), EMD-12787 (MS2-Random4, D5), EMD-12788 (MS2-Random4, D3-A), EMD-12789 (MS2-Random4, D3-B), EMD-12790 (MS2-SpyTag7, *T* = 3), EMD-12791 (MS2-SpyTag7, *T* = 4), EMD-12792 (MS2-SpyTag7, D5), EMD-12793 (MS2-SpyTag7, D3-A). Plasmids encoding the modified MS2 CP are available from the corresponding author upon reasonable request.

## Code availability

Custom code used for modeling the assembly process is available for download via the authors’ GitHub page: https://github.com/MathematicalComputationalVirology/MS2Nanotechnology
