# Supplementary material for: Programmable polymorphism of a virus-like particle
Source: Commun Mater. Author manuscript; Available in PMC 2022 Mar 11. (PMC7612486; doi:10.1038/s43246-022-00229-3)
Supplement: Supplementary Materials [file EMS143810-supplement-Supplementary_Materials.pdf]

Supplementary Materials for

Programmable Polymorphism of a Virus-Like Particle

Artur P. Biela‡, Antonina Naskalska‡, Farzad Fatehi, Reidun Twarock, Jonathan G. Heddle*

*Email: jonathan.heddle@uj.edu.pl

This PDF file includes:

Supplementary Discussion

Supplementary Methods

Tables S1 to S13

References (1 to 2)

Supplementary Discussion

Ensembles of particle morphologies compatible with a given dimer ratio.

Given a fixed number *N(AB)* of A/B dimers and *N(CC)* of C/C dimers, it is possible to construct distinct ensembles of particle morphologies from a geometrical point of view. Calling α, β, γ, δ, and ε the numbers of *T*=3, D5, *T*=4, D3-A, and D3-B particles in the ensemble, we obtain the relations $60(\alpha + \beta + \gamma + \delta + \varepsilon) = N(AB)$, and $30\alpha + 45\beta + 60\gamma + 51\delta + 57\varepsilon = N(CC)$. For SpyTag we have δ = ε = 0. Using the experimentally determined values *N(AB)*=6 883,200 and *N(CC)*=4 105,590 (see **Supplementary Table 4**), we obtain $\alpha = 92587 - \beta/2$, and $\gamma = 22{,}133 - \beta/2$. From a purely geometric point of view, any value β, i.e., the number of D5 particles in the ensemble, between 0 and 44,266 is possible. The value β=0 would favor the formation of icosahedrally symmetric *T*=3 and *T*=4 particles over lower-symmetry D5 particles. However, the number β=10,456 seen in the experiment implies that the symmetry of the final products is not the only determinant of particle morphology. A purely thermodynamic argument is therefore not sufficient, and aspects of assembly kinetics must also be considered.

In order to assess this effect qualitatively for different SpyTag options, we compare the maximal number of *T*=3 particles that could be built from the building blocks in the final ensemble by dissociating lower symmetry species and building higher symmetry particles from their constituent building blocks. The latter is a proxy for assembly given an idealised "instantaneous" conversion rate and can be used to benchmark against the particle numbers observed experimentally based on the actual conversion rate (**Figure 1e**). For SpyTag, this means comparing the β=0 case with the β=10456 scenario observed (**Figure 1c**). For all other scenarios, there are two reductions that need to be considered, first converting all D3-A particles into D5 and D3-B particles (δ=0) and then converting all D5 particles into *T*=3 and *T*=4 particles (β=0) (**Figure 1d**). As a result, the percentage of *T*=3 particles in the ensemble increases in this "ideal" scenario (**Supplementary Table 8**), and the increase is an indicator of kinetic contributions to the assembly outcome. Our kinetic assembly model quantifies such contributions and demonstrates the importance of the conversion rate from the symmetric C/C to the asymmetric A/B dimer for the experimental outcome.

Supplementary Methods

MS2 CP genetic constructs. Nucleotide sequences of generated CP variants were obtained from a synthetic gene provider (MS2-SpyTag) or sequencing service provider (MS2-SpyTag4, MS2-SpyTag7, MS2-Random). SpyTag sequence is represented in red, KpnI restriction site in green, linker sequences in blue.

- MS2-SpyTag (synthetic sequence):

ATGGCTTCTAACTTTACTCAGTTCGTTCTCGTCGACAATGGCGGAACTGGCGACGTGACTGTCGCCCCAAGCAACTT
CGCTAACGGGGTCGCTGAATGGATCAGCTCTAACTCGCGTTCACAGGCTTACAAAGTAACCTGTAGCGTTCGTCAG
AGCTCTGCGCAGAATCGCAAATACACCATCAAAGTCGAGGTGCCTAAAGTGGCAACCCAGACTGTTGGTGGTGTA
GAGCTTCCTGTAGCCGCATGGCGTTCGTACTTAAATATGGAACTAACCATTCCAATTTTCGCTACGAATTCCGACTG
CGAGCTTATTGTTAAGGCAATGCAAGGTCTCCTAAAAGATGGAAACCCGATTCCCTCAGCAATCGCAGCAAACTCC
GGCATCTACGCTAACTTTACTCAGTTCGTTCTCGTCGACAATGGCGGTACCGCTCATATTGTTATGGTTGATGCTTAC
AAGCCAACTAAGGGTACCGGCGACGTGACTGTCGCCCCAAGCAACTTCGCTAACGGGGTCGCTGAATGGATCAGC
TCTAACTCGCGTTCACAGGCTTACAAAGTAACCTGTAGCGTTCGTCAGAGCTCTGCGCAGAATCGCAAATACACCAT
CAAAGTCGAGGTGCCTAAAGTGGCAACCCAGACTGTTGGTGGTGTAGAGCTTCCTGTAGCCGCATGGCGTTCGTA
CTTAAATATGGAACTAACCATTCCAATTTTCGCTACGAATTCCGACTGCGAGCTTATTGTTAAGGCAATGCAAGGTC
TCCTAAAAGATGGAAACCCGATTCCCTCAGCAATCGCAGCAAACTCCGGCATCTACTAA

- MS2-SpyTag4:

ATGGCTTCTAACTTTACTCAGTTCGTTCTCGTCGACAATGGCGGAACTGGCGACGTGACTGTCGCCCCAAGCAACTT
CGCTAACGGGGTCGCTGAATGGATCAGCTCTAACTCGCGTTCACAGGCTTACAAAGTAACCTGTAGCGTTCGTCAG
AGCTCTGCGCAGAATCGCAAATACACCATCAAAGTCGAGGTGCCTAAAGTGGCAACCCAGACTGTTGGTGGTGTA
GAGCTTCCTGTAGCCGCATGGCGTTCGTACTTAAATATGGAACTAACCATTCCAATTTTCGCTACGAATTCCGACTG
CGAGCTTATTGTTAAGGCAATGCAAGGTCTCCTAAAAGATGGAAACCCGATTCCCTCAGCAATCGCAGCAAACTCC
GGCATCTACGCTAACTTTACTCAGTTCGTTCTCGTCGACAATGGCGGTACCGGCGGCGGCAGCGCTCATATTGTTAT
GGTTGATGCTTACAAGCCAACTAAGGGCGGCGGCAGCGGTACCGGCGACGTGACTGTCGCCCCAAGCAACTTCGC
TAACGGGGTCGCTGAATGGATCAGCTCTAACTCGCGTTCACAGGCTTACAAAGTAACCTGTAGCGTTCGTCAGAGC
TCTGCGCAGAATCGCAAATACACCATCAAAGTCGAGGTGCCTAAAGTGGCAACCCAGACTGTTGGTGGTGTAGAG
CTTCCTGTAGCCGCATGGCGTTCGTACTTAAATATGGAACTAACCATTCCAATTTTCGCTACGAATTCCGACTGCGA
GCTTATTGTTAAGGCAATGCAAGGTCTCCTAAAAGATGGAAACCCGATTCCCTCAGCAATCGCAGCAAACTCCGGC
ATCTACTAA

- MS2-SpyTag7:

ATGGCTTCTAACTTTACTCAGTTCGTTCTCGTCGACAATGGCGGAACTGGCGACGTGACTGTCGCCCCAAGCAACTT
CGCTAGCGGGGTCGCTGAATGGATCAGCTCTAACTCGCGTTCACAGGCTTGCAAAGTAACCTGTAGCGTTCGTCAG
AGCTCTGCGCAGAATCGCAAATACGCCATCAAAGTCGAGGTGCCTAAAGTGGCAACCCAGACTGTTGGTGGTGTA
GAGCTTCCTGTAGCCGCATGGCGTTCGTACTTAAATATGGAACTAACCATTCCAATTTTCGCTACGAATTCCGACTG
CGAGCTTATTGTTAAGGCAATGCAAGGTCTCCTAAAAGATGGAAACCCGATTCCCTCAGCAATCGCAGCAAACTCC
GGCATCTACGCTAACTTTACTCAGTTCGTTCTCGTCGACAATGGCGGTACCGGTGGTAGTGGCGGCGGCAGCGCTC
ATATTGTTATGGTTGATGCTTACAAGCCAACTAAGGGCGGCGGCAGCGGTGGTAGTGGTACCGGCGACGTGACTG
TCGCCCCAAGCAACTTCGCTAACGGGGTCGCTGAATGGATCAGCTCTAACTCGCGTTCACAGGCTTACAAAGTAAC
CTGTAGCGTTCGTCAGAGCTCTGCGCAGAATCGCAAATACACCATCAAAGTCGAGGTGCCTAAAGTGGCAACCCAG
ACTGTTGGTGGTGTAGAGCTTCCTGTAGCCGCATGGCGTTCGTACTTAAATATGGAACTAACCATTCCAATTTTCGC
TACGAATTCCGACTGCGAGCTTATTGTTAAGGCAATGCAAGGTCTCCTAAAAGATGGAAACCCGATTCCCTCAGCA
ATCGCAGCAAACTCCGGCATCTACTAA

- MS2-Random4:

ATGGCTTCTAACTTTACTCCGTTCGTTCTCGTCGACAATGGCGGAACTGGCGACGTGACTGTCGCCCCAAGCAACTT
CGCTAACGGGGTCGCTGAATGGATCAGCTCTAACTCGCGTTCACAGGCTTACAAAGTAACCTGTAGCGTTCGTCAG
AGCTCTGCGCAGAATCGCAAATACACCATCAAAGTCGAGGTGCCTAAAGTGGCAACCCAGACTGTTGGTGGTGTA
GAGCTTCCTGTAGCCGCATGGCGTTCGTACTTAAATATGGAACTAACCATTCCAATTTTCGCTACGAATTCCGACTG
CGAGCTTATTGTTAAGGCAATGCAAGGTCTCCTAAAAGATGGAAACCCGATTCCCTCAGCAATCGCAGCAAACTCC
GGCATCTACGCTAACTTTACTCAGTTCGTTCTCGTCGACAATGGCGGTACCGGCGGCGGCAGCTACGCTACTATGC
CAATTGCTAAGCATGTTAAGGATGTTGGCGGCGGCAGCGGTACCGGCGACGTGACTGTCGCCCCAAGCAACTTCG
CTAACGGGGTCGCTGAATGGATCAGCTCTAACTCGCGTTCACAGGCTTACAAAGTAACCTGTAGCGTTCGTCAGAG
CTCTGCGCAGAATCGCAAATACACCATCAAAGTCGAGGTGCCTAAAGTGGCAACCCAGACTGTTGGTGGTGTAGA
GCTTCCTGTAGCCGCATGGCGTTCGTACTTAAATATGGAACTAACCATTCCAATTTTCGCTACGAATTCCGACTGCG
AGCTTATTGTTAAGGCAATGCAAGGTCTCCTAAAAGATGGAAACCCGATTCCCTCAGCAATCGCAGCAAACTCCGG
CATCTACTAA

Supplementary Figures

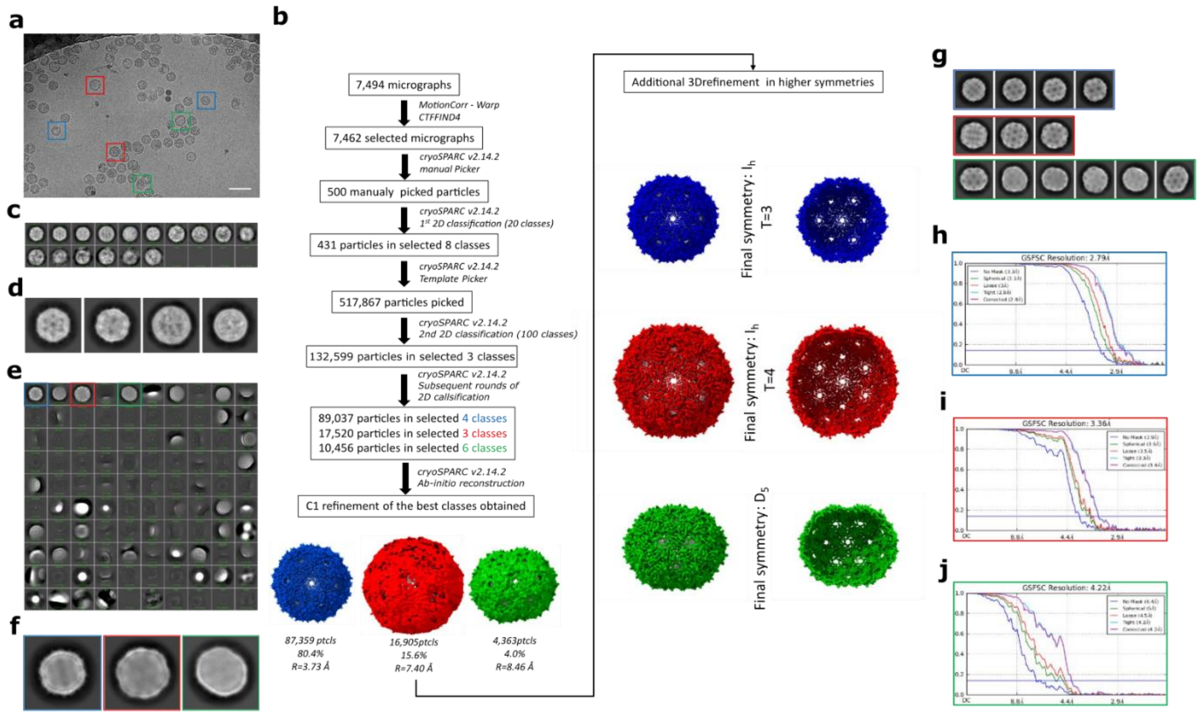

Supplementary Figure 1. Procedure for cryo-EM reconstruction of MS2-SpyTag sample: **a** Representative micrograph with three different particles marked in boxes (f ~ 26 nm, f ~ 34 nm and non-spherical in blue, red and green respectively); Scale bar – 50 nm. **b** Summary of the image processing procedure (see Methods). **c** Initial 2D class averages. **d** Selected 2D class averages used for template search. **e** Reference free 2D classification after template pick. **f** Selected 3 different 2D class averages. **g,** final 2D class averages for each of the identified particles. **h-j** FSC correlation curves for each of the solved densities after applying highest possible symmetry.

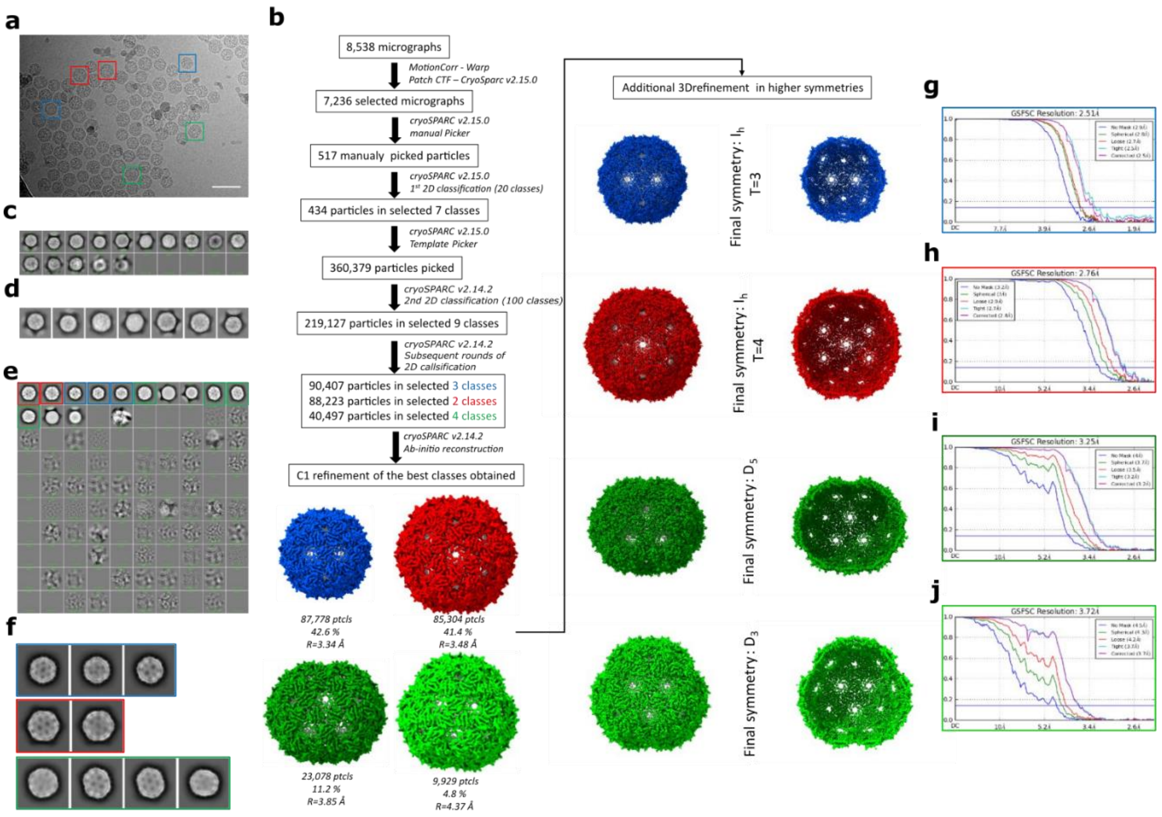

Supplementary Figure 2. Procedure for cryo-EM reconstruction of MS2-SpyTag4 sample: **a** Representative micrograph with three different particles marked in boxes (f ~ 26 nm, f ~ 34 nm and non-spherical in blue, red and green respectively); Scale bar – 50 nm. **b** Summary of the image processing procedure (see Methods). **c** Initial 2D class averages. **d** Selected 2D class averages used for template search. **e** Reference free 2D classification after template pick. **f** Final 2D class averages for each of the identified particles. **g-j** FSC correlation curves for each of the solved densities after applying highest possible symmetry.

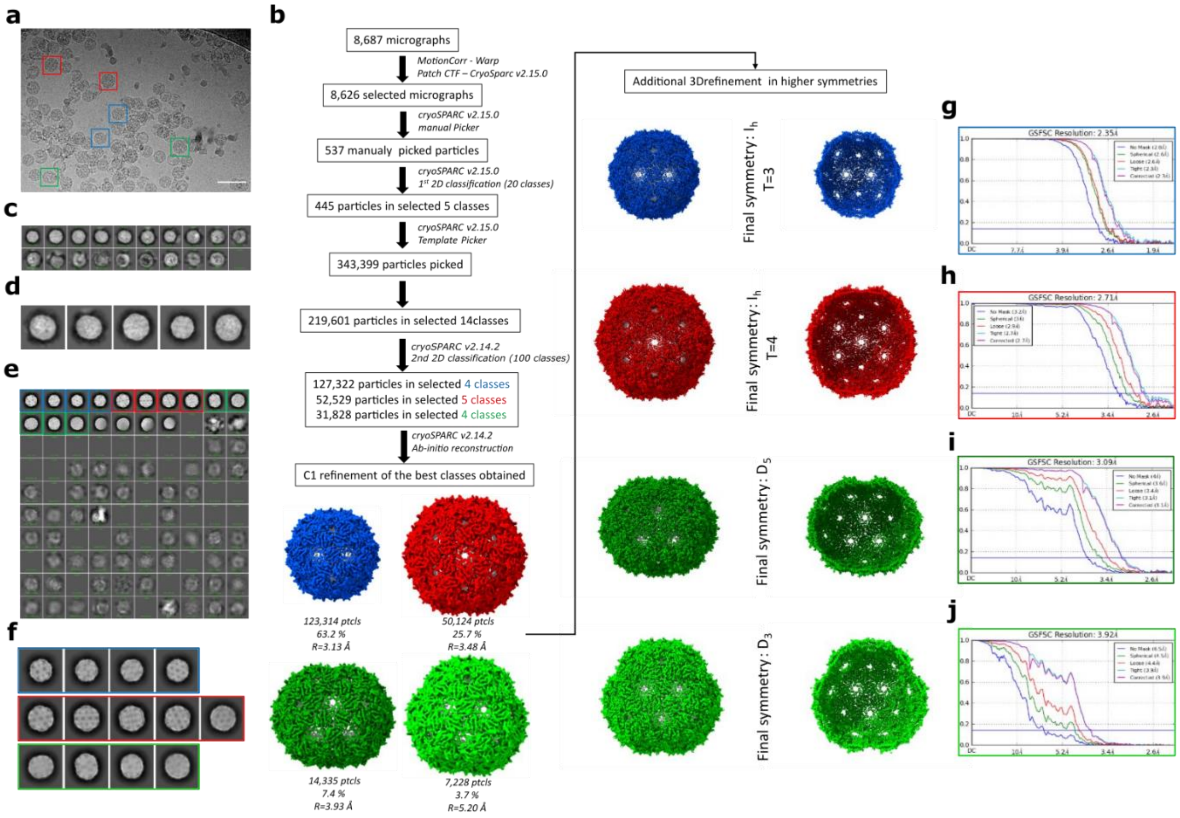

Supplementary Figure 3. Procedure for cryo-EM reconstruction of MS2-SpyTag7 sample: **a** Representative micrograph with three different particles marked in boxes (f ~ 26 nm, f ~ 34 nm and non-spherical in blue, red and green respectively); Scale bar – 50 nm. **b** Summary of the image processing procedure (see Methods). **c** Initial 2D class averages. **d** Selected 2D class averages used for template search. **e** Reference free 2D classification after template pick. **f** Final 2D class averages for each of the identified particles. **g-j** FSC correlation curves for each of the solved densities after applying highest possible symmetry.

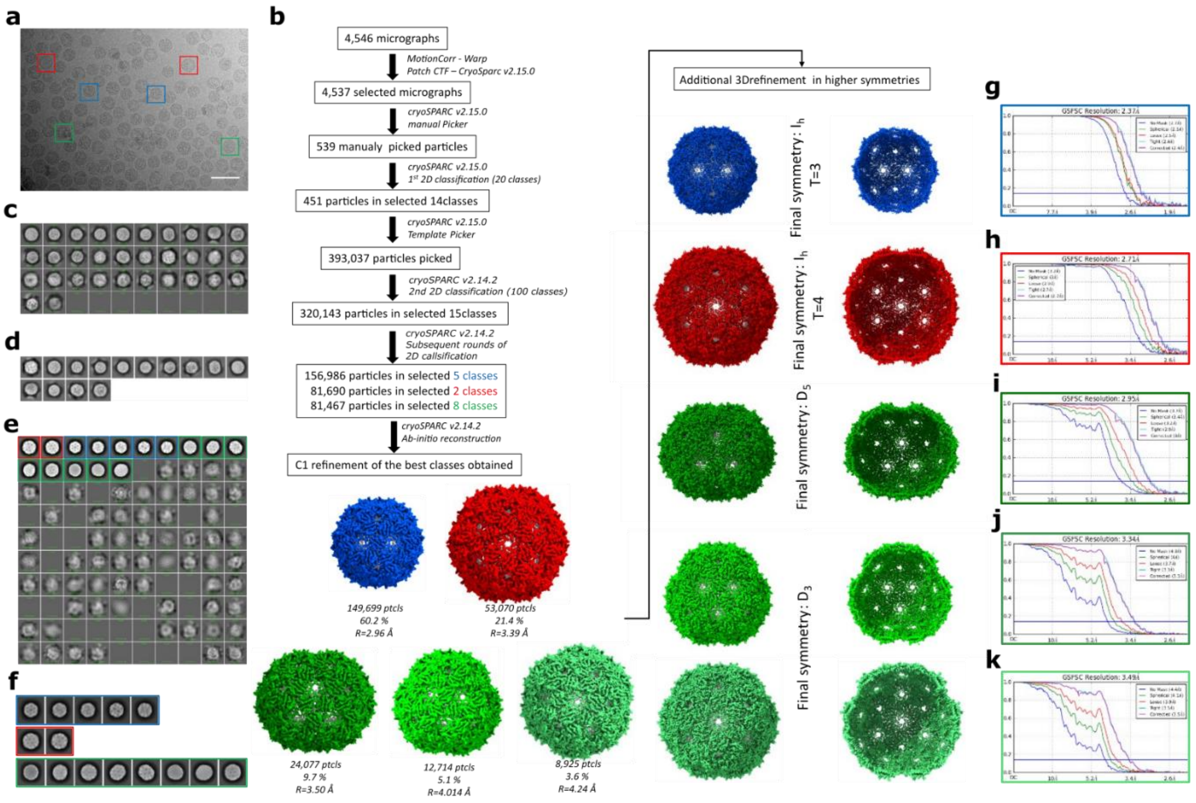

Supplementary Figure 4. Procedure for cryo-EM reconstruction MS2-Random4 sample: **a** Representative micrograph with three different particles marked in boxes (f ~ 26 nm, f ~ 34 nm and non-spherical in blue, red and green respectively); Scale bar – 50 nm. **b** Summary of the image processing procedure (see Methods). **C** Initial 2D class averages. **d** Selected 2D class averages used for template search. **e,** reference free 2D classification after template pick. **f** Final 2D class averages for each of the identified particles. **g-k,** FSC correlation curves for each of the solved densities after applying highest possible symmetry.

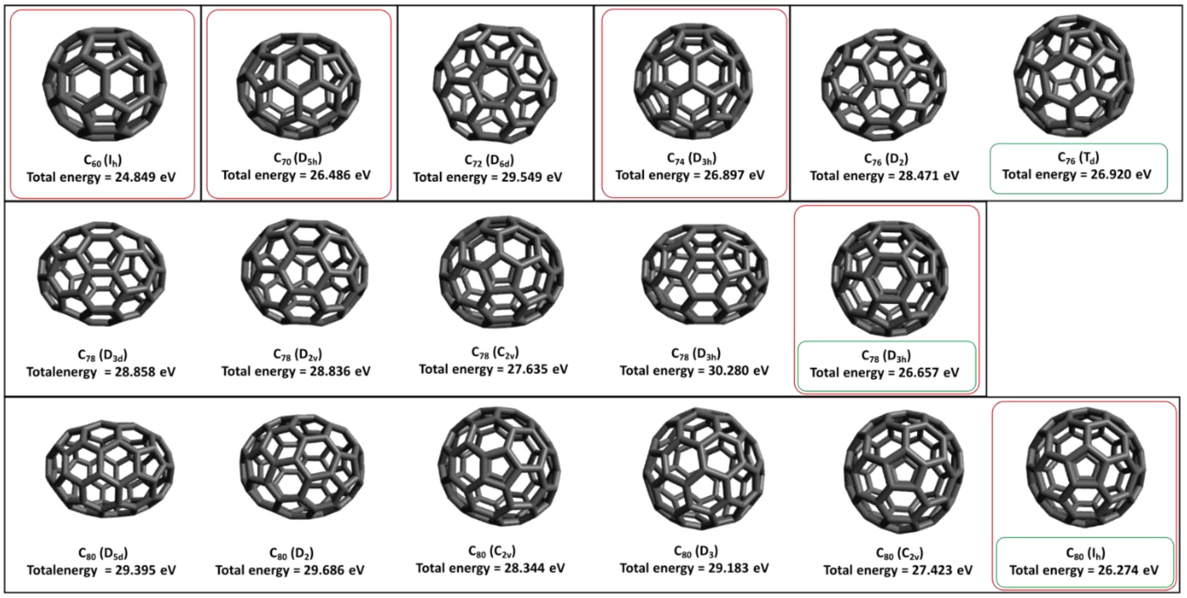

Supplementary Figure 5. Structures of fullerenes with carbon atom numbers from 60 to 80 with associated symmetries and total strain energies[1]**:** When isomers exist, those with lowest strain energies are marked with green frame; geometries identified in corresponding MS2 VLPs variants marked with red frames. Figure prepared using Avogadro[2].

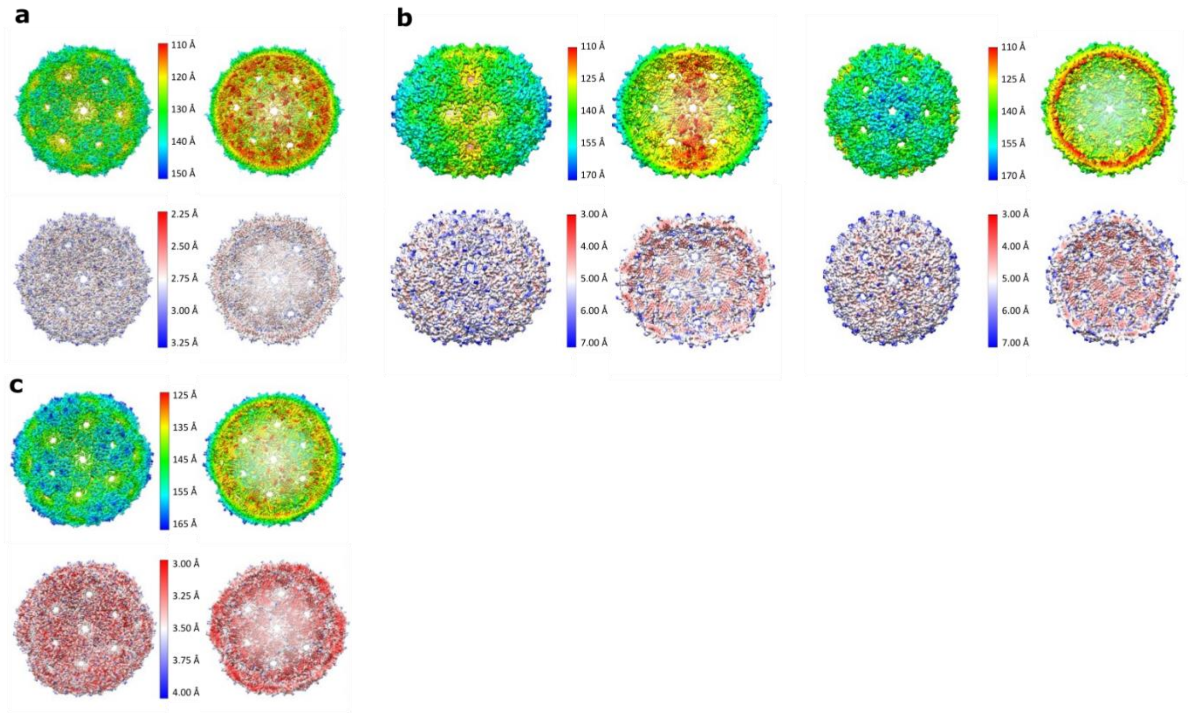

Supplementary Figure 6. MS2-SpyTag VLPs showed in radius colouring scheme (top) and local resolution (bottom): **a** *T*=3 particle. **b** D5 particle; two orthogonal views are shown (left and right). **c** *T*=4 particle. Every structure is shown in external surface view (left) as well as a cross section revealing inner surface of the VLPs (right).

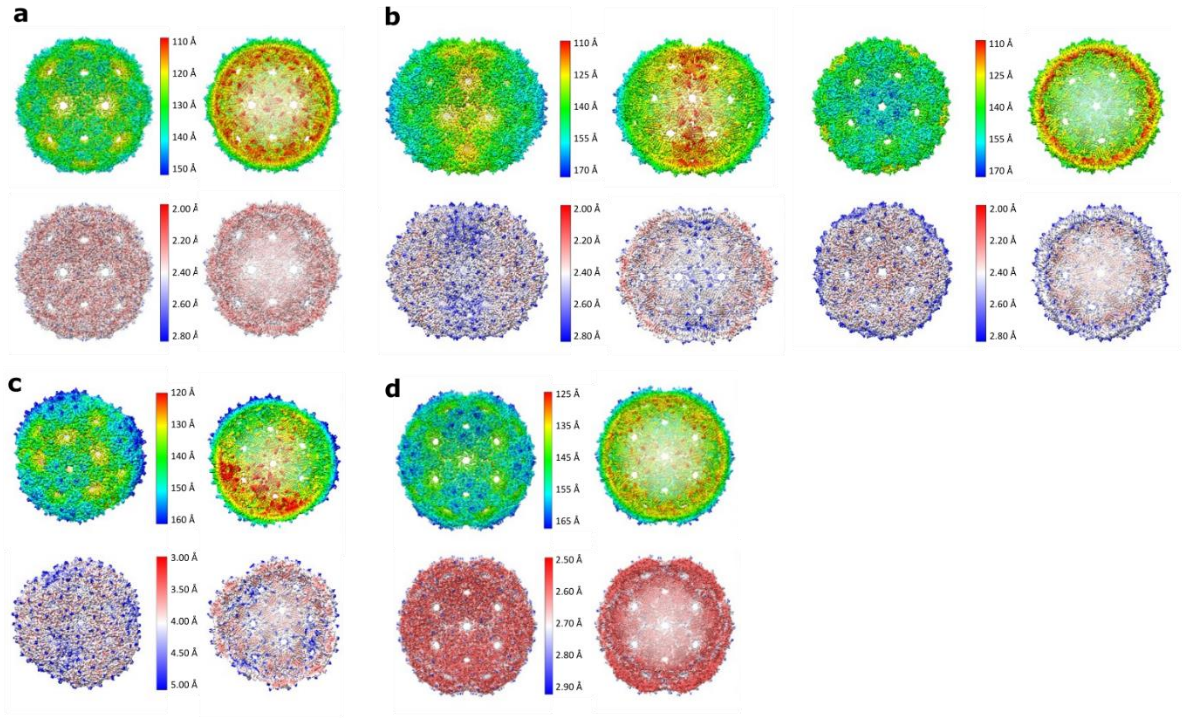

Supplementary Figure 7. MS2-SpyTag4 VLPs showed in radius colouring scheme (top) and local resolution (bottom): **a** *T*=3 particle. **b** D5 particle; two orthogonal views are shown (left and right). **c** D3-A particle. **d** *T*=4 particle. Every structure is shown in external surface view (left) as well as a cross section revealing inner surface of the VLPs (right).

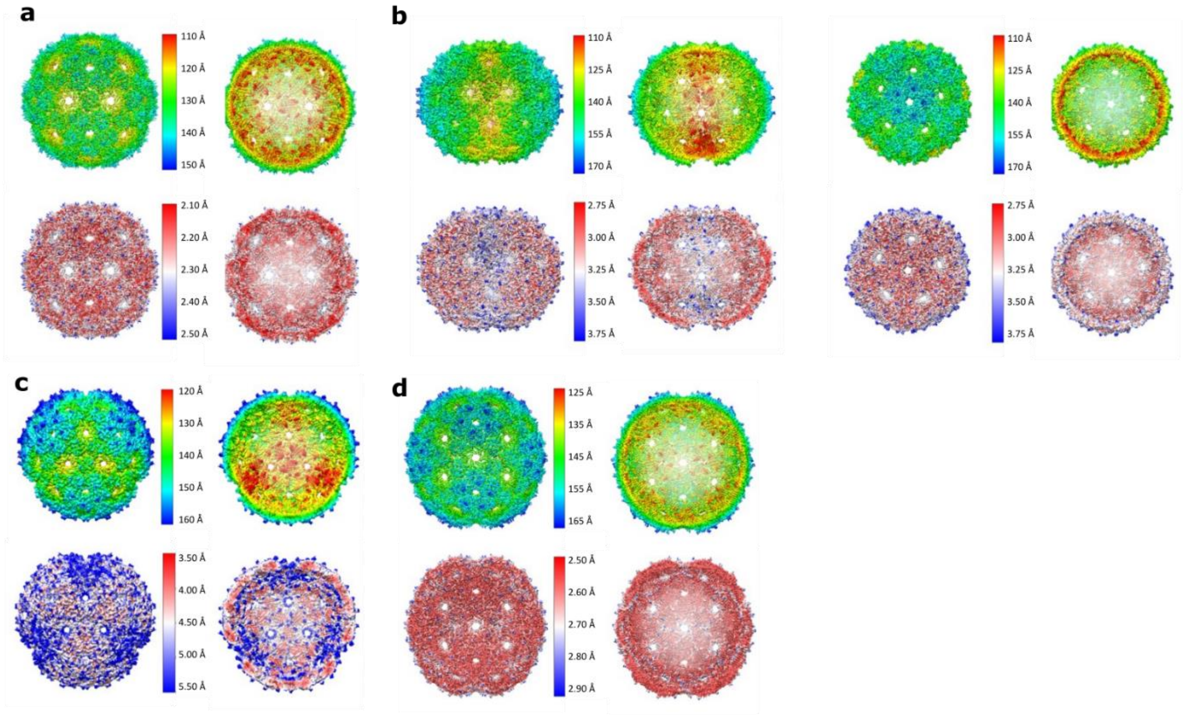

Supplementary Figure 8. MS2-SpyTag7 VLPs showed in radius colouring scheme (top) and local resolution (bottom): **a** *T*=3 particle. **b** D5 particle; two orthogonal views are shown (left and right). **c** D3-A particle. **d** *T*=4 particle. Every structure is shown in external surface view (left) as well as a cross section revealing inner surface of the VLPs (right).

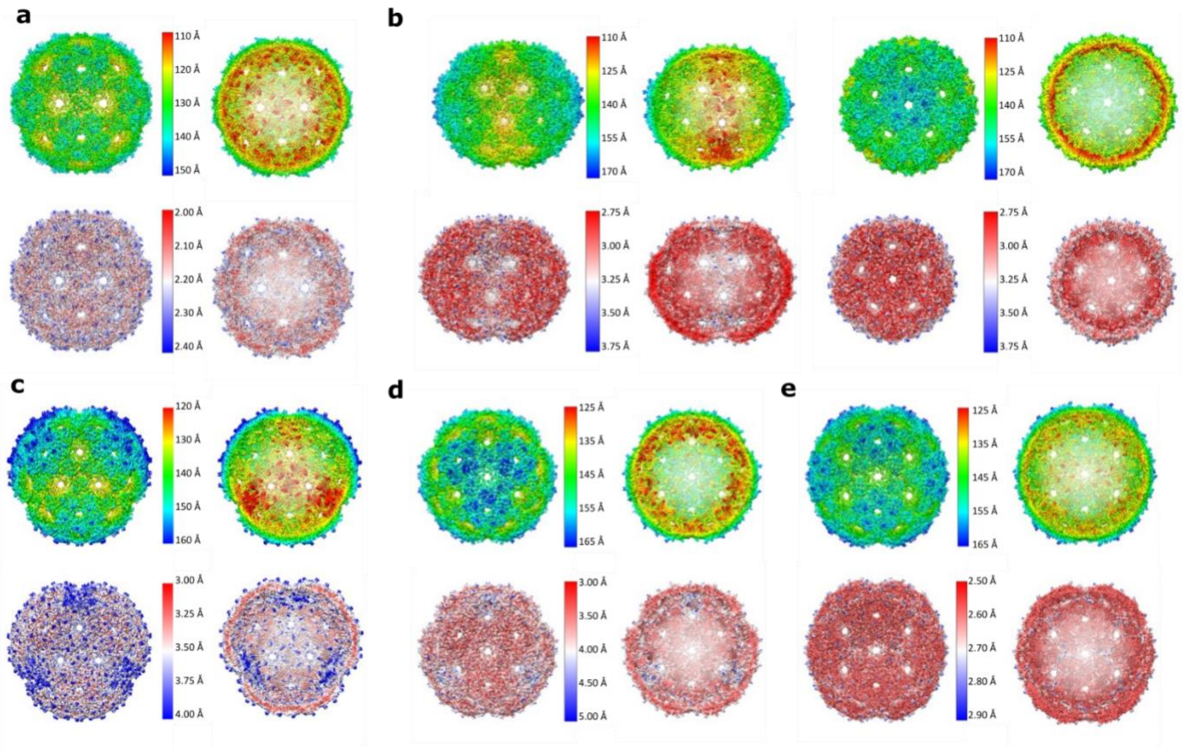

Supplementary Figure 9. MS2-Random4 VLPs showed in radius colouring scheme (top) and local resolution (bottom): **a** *T*=3 particle. **b** D5 particle; two orthogonal views are shown (left and right). **c** D3-A particle. **d** D3-B particle. **e** *T*=4 particle. Every structure is shown in external surface view (left) as well as a cross section revealing inner surface of the VLPs (right).

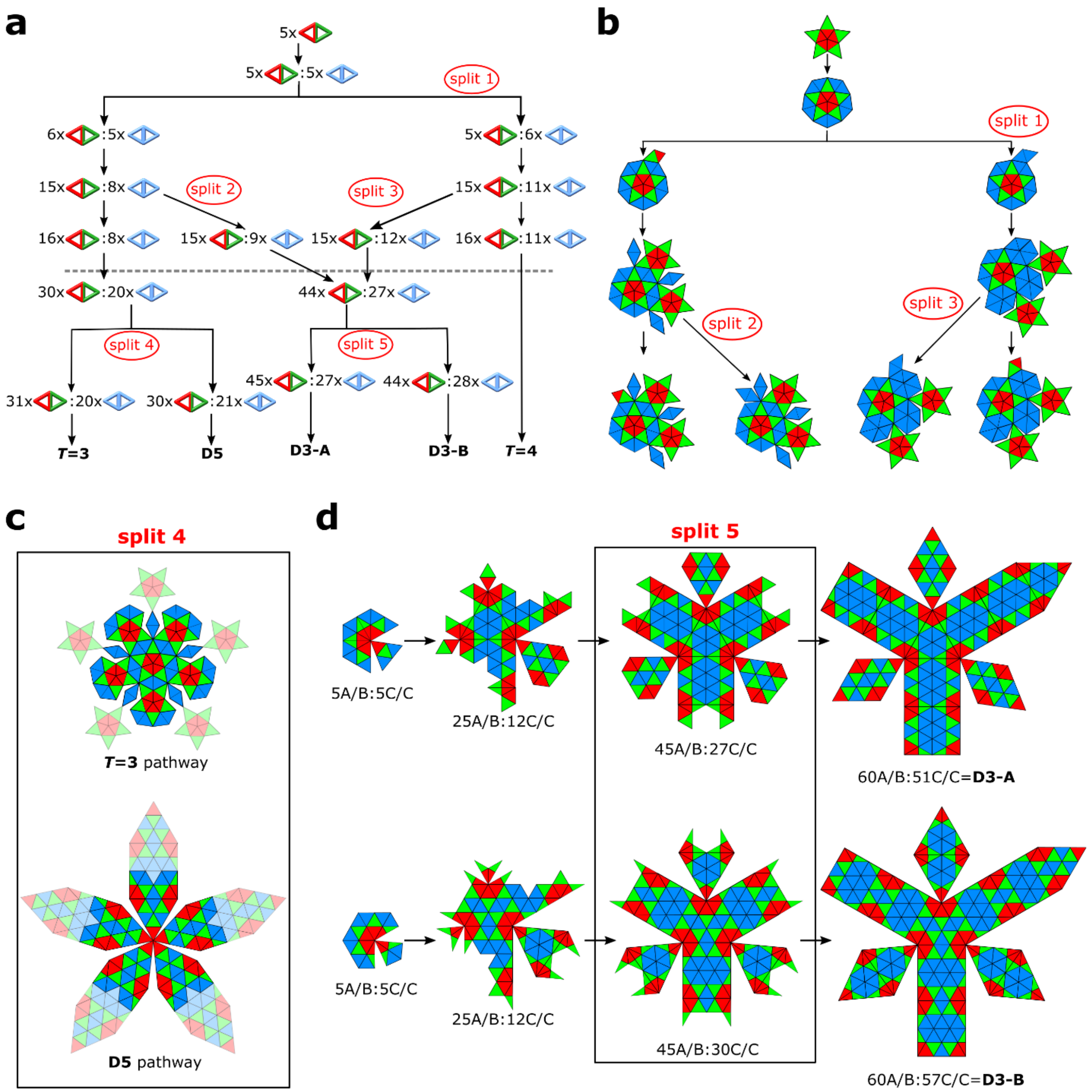

Supplementary Figure 10. The bifurcation of assembly pathways and intermediate geometries shared by different particle types: **a** Diagrammatic illustration of bifurcation points ("splits") in the assembly pathways leading to different particle types. **b** Tiling representation of the intermediate particle geometries at bifurcation points in the assembly pathways above the grey dashed line in a, with green/red and blue/blue rhomboids indicating A/B and C/C dimers, respectively. **c** Tiling model of the particle geometry at split point 4, showing the next dimers to be recruited on the pathway towards a *T*=3 (top) and D5 particle (bottom) in faint colours. In particular, the *T*=3 pathway must next acquire an A/B dimer (green/red), while the D5 pathway must recruit a C/C dimer (blue/blue). **d** Shows assembly intermediates on an assembly pathway of D3-A and D3-B particles, illustrating the geometry of the shared intermediate at split point 5.

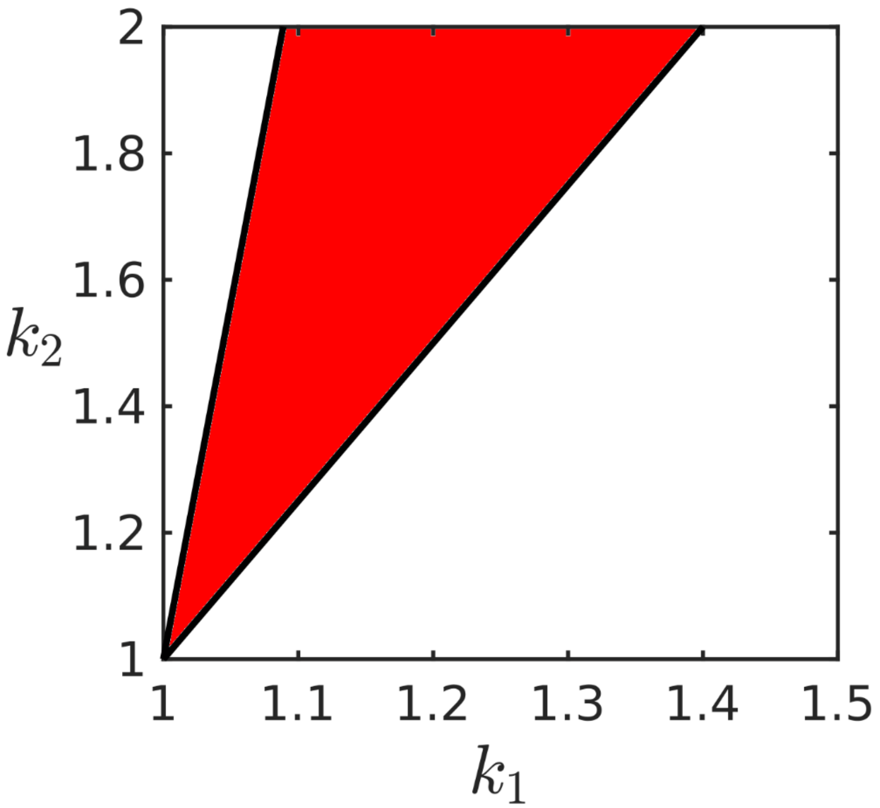

Supplementary Figure 11. The parameter space describing elastic contributions from different C/C dimer positions. The elastic energy of each particle depends on the bending and stretching of subunits in different positions and is parametrised by k_1 and k_2. For parameters in the red area the abundance of different particle types corresponds to the rank order seen in the experiments.

Uncropped gels shown in Fig. 2b

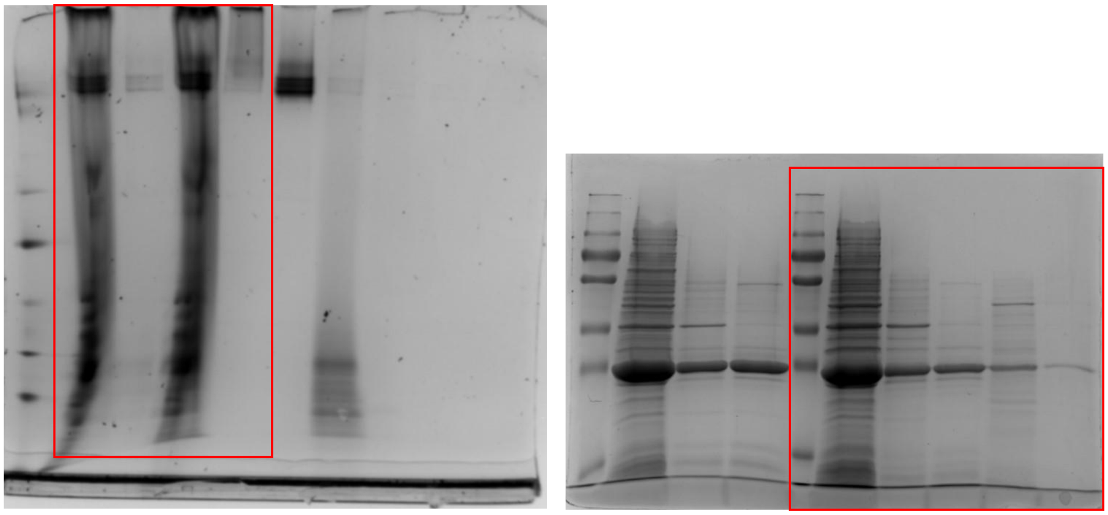

Uncropped gel shown in Fig. 3a

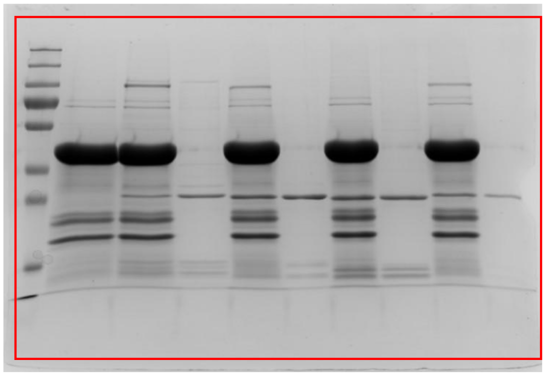

Supplementary Figure 12. Entire images of gels shown in Figures 2b and 3a of the main text. Frames indicate cropped areas.

Uncropped versions of the TEM images shown in Fig. 2d

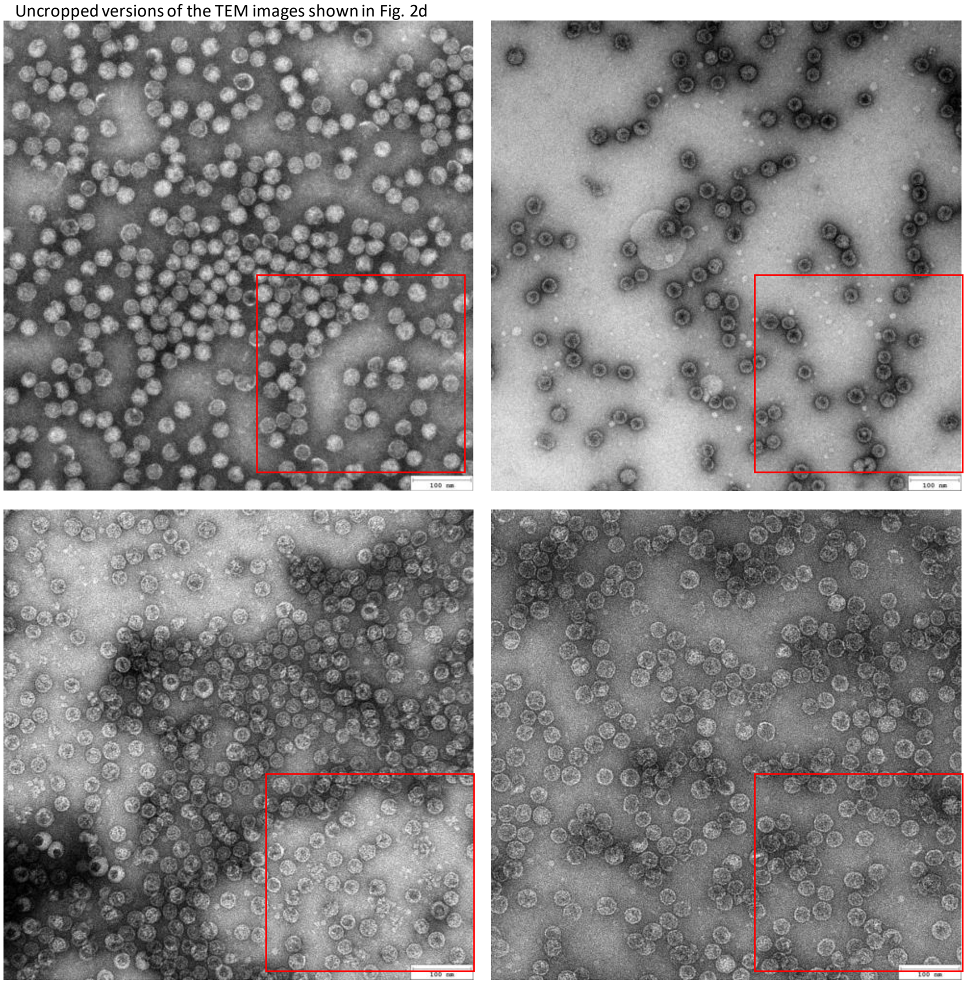

Uncropped version of the TEM image shown in Fig. 3d

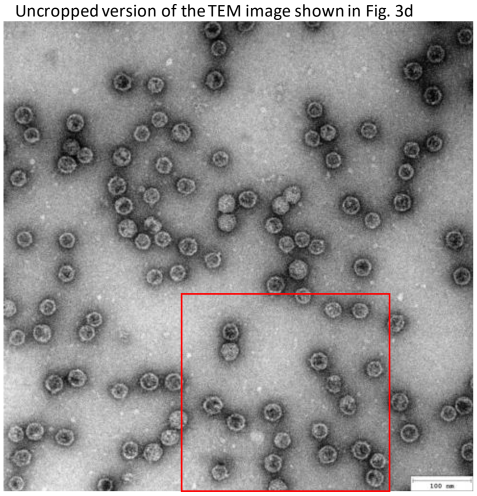

Supplementary Figure 13. Entire TEM images shown in Figures 2d and 3d of the main text. Frames indicate cropped areas.

Supplementary Tables

Supplementary Table 1. Primers used to generate SpyTag 4aa linkers; Spytag 7aa linkers and Random Peptide 4aa linkers insertions into the CP gene.

SpyTag 4 aa Fwd	5' CTGAGGTACCGGCGGCGGCAGCGCTCATATTGTTATGG
SpyTag 4 aa Rev	5' CTGAGGGTACCGCTGCCGCCGCCCTTAGTTGGCTTG
SpyTag 7aa Fwd	5' cttGGTACCggtggtagtggcggcggcagcgctcat
SpyTag 7aa Rev	5' actaagggcggcggcagcggtggtagtggtaccaag
Random 4 aa Fwd	5' ctgaggtaccggcggcggcagctacgctactatgccaattgctaagcatg
Random 4 aa Rev	5' ctgaggtaccgctgccgccgccaacatccttaacatgcttagcaattggc

Supplementary Table 2. Structures of identified MS2-SpyTag VLPs – summary

**Sample**	**MS2-SpyTag**			**MS2-SpyTag4**				**MS2-SpyTag7**				**Random4**				
**Symmetry**	*T=3*	D5	*T=4*	*T=3*	D_5	D3 (D3-A)	*T=4*	*T=3*	D_5	D3 (D3-A)	*T=4*	*T=3*	D_5	D3 (D3-A)	D3 (D3-B)	*T=4*
**Resolution [Å] $FSC_{0.143}$**	2.79	4.22	3.36	2.51	3.25	3.72	2.76	2.35	3.09	3.92	2.71	2.37	2.95	3.34	3.49	2.71
**EMD-id**	EMD-12778	EMD-12779	EMD-12780	EMD-12781	EMD-12782	EMD-12783	EMD-12784	EMD-12785	EMD-12786	EMD-12787	EMD-12788	EMD-12789	EMD-12790	EMD-12791	EMD-12792	EMD-12793

Supplementary Table 3. Categorization of particle types into constituent numbers of A/B and C/C dimers.

Particle type	*T*=3	D5	*T*=4	D3-A	D3-B
A/B	60	60	60	60	60
C/C	30	45	60	51	57

Supplementary Table 4. Number of A/B dimers in all discovered geometries of MS2 VLP variants; [a] – experimental data

sample	MS2-SpyTag			MS2-SpyTag4				MS2-SpyTag7				Random4				
symmetry	*T=3*	D5	*T=4*	*T=3*	D5	D3-A	*T=4*	*T=3*	D5	D3-A	*T=4*	*T=3*	D5	D3-A	D3-B	*T=4*
A/B dimers	60	60	60	60	60	60	60	60	60	60	60	60	60	60	60	60
C/C dimers	30	45	60	30	45	51	60	30	45	51	60	30	45	51	57	60
no. of identified particles[a]	87,359	10,456	16,905	87,778	23,078	9,929	85,304	123,314	14,135	7,228	50,124	149,699	24,077	12,714	8,925	53,070
total no. of identified particles[a]	114,720			206,089				194,801				248,485				
total no of A/B dimers[a]	5 241,540	627,360	1 014,300	5 266,680	1 384,680	595,740	5 118,240	7 398,840	848,100	433,680	3 007,440	8 981,940	1 444,620	762,840	535,500	3 184,200
total no of C/C dimers[a]	2 620,770	470,520	1 014,300	2 633,340	1 038,510	506,379	5 118,240	3 699,420	636,075	368,628	3 007,440	4 490,970	1 083,465	648,414	508,725	3 184,200
A/B dimers:all dimers ratio[a]	0.667	0.571	0.500	0.667	0.571	0.541	0.500	0.667	0.571	0.541	0.500	0.667	0.571	0.541	0.513	0.500
total A/B dimers:all dimers ratio[a]	0.626			0.571				0.602				0.601				

Supplementary Table 5. Estimated values of parameters. *f* is the conversion from C/C to A/B, with backward rate *b* which is assumed to be fixed. *Split k*, *k*=1 to 5, refer to nodes in the assembly graph in Fig. 1, where the pathways to different geometries bifurcate. f^{cc}_{elong} and f^{ab}_{elong} are the rates at which C/C and A/B dimers bind after the nucleation step (5A/B+5C/C), respectively.

parameters	SpyTag	SpyTag4	SpyTag7	Random4
f	5.2 S^{-1}	0.29 S^{-1}	1.5 S^{-1}	1.64 S^{-1}
b	0.02 S^{-1}	0.02 S^{-1}	0.02 S^{-1}	0.02 S^{-1}
Split 1	0.14	0.28	0.14	0.14
Split 2	0.022	0.022	0.022	0.022
Split 3	0.031	0.031	0.031	0.031
Split 4	0.07	0.11	0.07	0.07
Split 5	0.11	0.11	0.11	0.11
f^{cc}_{elong}	$10^6\ M^{-1}S^{-1}$	$0.7 \times 10^6\ M^{-1}S^{-1}$	$10^6\ M^{-1}S^{-1}$	$10^6\ M^{-1}S^{-1}$
f^{ab}_{elong}	$10^6\ M^{-1}S^{-1}$	$10^6\ M^{-1}S^{-1}$	$10^6\ M^{-1}S^{-1}$	$10^6\ M^{-1}S^{-1}$

Supplementary Table 6. The ratios for different particle types with respect to the *T*=3 particles as a function of f. This indicates that by increasing the conversion rate f the number of other particles with respect to the number of *T*=3 (wild type) particles reduce. This shows that inclusion of a tag to C/C dimers will reduce the conversion rate and leads to the formation of D5, D3, and *T*=4 particles.

Particle / *f*	D5	D3-A	D3-B	*T*=4
10 S^{-1}	0.06	0.02	0.003	0.1
15 S^{-1}	0.04	0.02	0.001	0.09
20 S^{-1}	0.03	0.01	0.0009	0.07
30 S^{-1}	0.02	0.009	0.0005	0.05
40 S^{-1}	0.02	0.007	0.0003	0.04

Supplementary Table 7. Ratios computed based on experimental data are shown on the left which have been used for model fitting and the model results are shown on the right.

	SpyTag		SpyTag4		SpyTag7		Random4	
	Experiment	Model	Experiment	Model	Experiment	Model	Experiment	Model
T=3	1	1	1	1	1	1	1	1
D5	0.12	0.1	0.26	0.27	0.11	0.16	0.16	0.15
D3-A	0	0.04	0.11	0.12	0.06	0.07	0.08	0.07
D3-B	0	0.007	0	0.05	0	0.02	0.06	0.02
T=4	0.19	0.2	0.97	0.93	0.41	0.38	0.35	0.36

Supplementary Table 8: Difference between the experimentally observed percentage of T=3 particles and the maximal number that could be obtained from the same building blocks in the overall ensemble when formation of the icosahedral T=3 and T=4 particles was favored.

	**SpyTag**	**SpyTag4**	**SpyTag7**	**Random4**
experiment	76.15%	43.8%	64.23%	60.24%
ideal scenario	80.85%	48.19%	66.93%	63.3%
% increase	4.70%	4.39%	2.7%	3.06%

References for Supplementary Figures

1 Tománek, D. Guide Through the Nanocarbon Jungle: Buckyballs. *Nanotubes, Graphene, and Beyond* (2014).

2 Hanwell, M. D. *et al.* Avogadro: an advanced semantic chemical editor, visualization, and analysis platform. *J. Cheminform.* **4**, 1-17 (2012).
